# Identification of MMP1 as a novel risk factor for intracranial aneurysms in ADPKD using iPSC models

**DOI:** 10.1038/srep30013

**Published:** 2016-07-15

**Authors:** Tomonaga Ameku, Daisuke Taura, Masakatsu Sone, Tomohiro Numata, Masahiro Nakamura, Fumihiko Shiota, Taro Toyoda, Satoshi Matsui, Toshikazu Araoka, Tetsuhiko Yasuno, Shin-Ichi Mae, Hatasu Kobayashi, Naoya Kondo, Fumiyo Kitaoka, Naoki Amano, Sayaka Arai, Tomoko Ichisaka, Norio Matsuura, Sumiko Inoue, Takuya Yamamoto, Kazutoshi Takahashi, Isao Asaka, Yasuhiro Yamada, Yoshifumi Ubara, Eri Muso, Atsushi Fukatsu, Akira Watanabe, Yasunori Sato, Tatsutoshi Nakahata, Yasuo Mori, Akio Koizumi, Kazuwa Nakao, Shinya Yamanaka, Kenji Osafune

**Affiliations:** 1 Center for iPS Cell Research and Application (CiRA), Kyoto University, Kyoto 606-8507, Japan; 2 Department of Medicine and Clinical Science, Kyoto University Graduate School of Medicine, Kyoto, 606-8507, Japan; 3 Department of Synthetic Chemistry and Biological Chemistry, Graduate School of Engineering, Kyoto University, Kyoto 615-8510, Japan; 4 Department of Technology and Ecology, Hall of Global Environmental Studies, Kyoto University, Kyoto 615-8510, Japan; 5 Department of Artificial Kidneys, Kyoto University Graduate School of Medicine, Kyoto 606-8507, Japan; 6 Department of Environmental and Health Sciences, Kyoto University School of Public Health, Kyoto 606-8501, Japan; 7 Division of Nephrology and Dialysis, Kitano Hospital The Tazuke Kofukai Medical Research Institute, Osaka 530-8480, Japan; 8 Institute for Integrated Cell-Material Sciences (iCeMS), Kyoto University, Kyoto 606-8501, Japan; 9 Nephrology Center and Okinaka Memorial Institute for Medical Research, Toranomon Hospital, Tokyo 105-8470, Japan; 10 Clinical Research Center, Chiba University of Medicine, Chiba 260-8677, Japan

## Abstract

Cardiovascular complications are the leading cause of death in autosomal dominant polycystic kidney disease (ADPKD), and intracranial aneurysm (ICA) causing subarachnoid hemorrhage is among the most serious complications. The diagnostic and therapeutic strategies for ICAs in ADPKD have not been fully established. We here generated induced pluripotent stem cells (iPSCs) from seven ADPKD patients, including four with ICAs. The vascular cells differentiated from ADPKD-iPSCs showed altered Ca^2+^ entry and gene expression profiles compared with those of iPSCs from non-ADPKD subjects. We found that the expression level of a metalloenzyme gene, matrix metalloproteinase (MMP) 1, was specifically elevated in iPSC-derived endothelia from ADPKD patients with ICAs. Furthermore, we confirmed the correlation between the serum MMP1 levels and the development of ICAs in 354 ADPKD patients, indicating that high serum MMP1 levels may be a novel risk factor. These results suggest that cellular disease models with ADPKD-specific iPSCs can be used to study the disease mechanisms and to identify novel disease-related molecules or risk factors.

Autosomal dominant polycystic kidney disease (ADPKD) is the most prevalent, potentially lethal, monogenic disorder, and is characterized by the development of multiple renal cysts and various extrarenal manifestations^1^,^2^. The disorder is caused by mutations to either of two genes, *PKD1* and *PKD2*, which encode polycystin-1 and -2, respectively^3^,^4^. Polycystin-1 is a plasma membrane receptor-like protein that has functions in cell-cell or cell-extracellular matrix interactions^5^. Polycystin-2 is a nonselective cation channel protein with high permeability for Ca^2+^ and interacts with polycystin-1 to form a functional receptor-ion channel complex that regulates intracellular Ca^2+^ homeostasis^6^,^7^,^8^,^9^,^10^. Patients with *PKD2* mutations show milder clinical phenotypes than those with *PKD1* mutations^11^.

Intracranial aneurysms (ICAs) are among the most serious cardiovascular complications in patients with ADPKD, since their rupture carries a severe morbidity-mortality rate of ~50%^12^. A prevalence of 8% for asymptomatic ICAs has been reported in patients with ADPKD, and those with *PKD1* and *PKD2* mutations appear to be at an equal risk of developing ICAs, while patients with mutations to the 5′ half of *PKD1* are more likely to develop ICAs^12^,^13^,^14^,^15^. Both polycystin-1 and -2 are expressed in the vascular endothelia and smooth muscle cells in humans and mice, and may play a role in the structural integrity of blood vessels^16^,^17^,^18^,^19^,^20^,^21^. Several mouse models were established to study the functional roles of the polycystins in vascular cells using targeted disruption of the *Pkd1* or *Pkd2* gene. It is likely that aberrant expression or localization of polycystins affects the fluid shear stress sensing in endothelia and the stretch-activated channel activity in smooth muscle cells, which results in altered intracellular Ca^2+^ homeostasis and the vascular phenotype associated with ADPKD^22^,^23^,^24^,^25^,^26^,^27^,^28^. However, little is known about the molecular mechanisms of ICA formation in ADPKD, and novel cellular assay systems are needed to study the mechanisms. In this study, we demonstrate the potential of using patient-derived iPSCs to create disease models and to identify novel risk factors for the vasculopathy associated with ADPKD.

## Results

### Generation of ADPKD-specific iPSCs from Patient Fibroblasts

**Figure 1:**
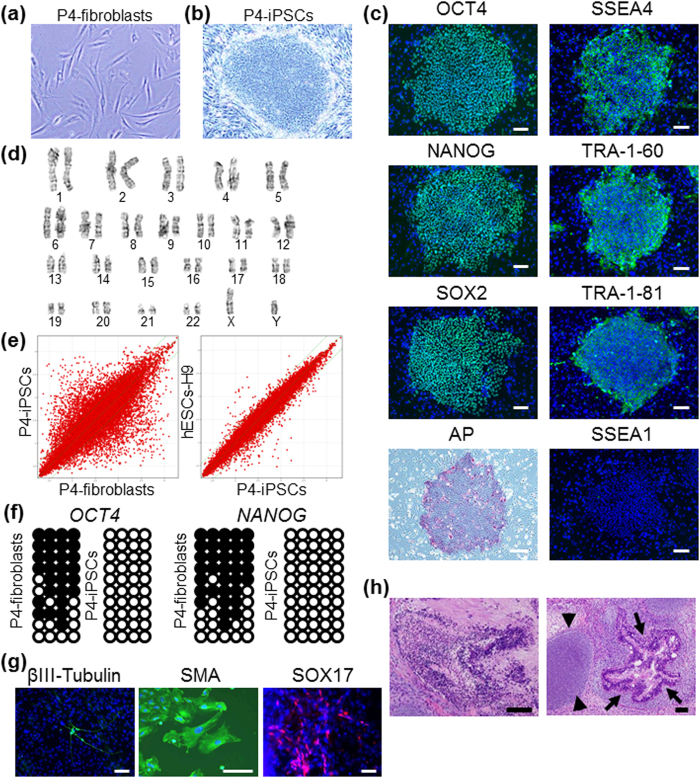
Derivation of Patient-specific iPSCs from ADPKD Patients. ADPKD patient’s (P4) fibroblasts (**a**) were converted into ADPKD-iPSCs (**b**) after retroviral transduction with OCT4, SOX2, KLF4 and c-MYC. (**c**) The expression of pluripotency markers, such as OCT4, NANOG, SOX2, SSEA4, TRA-1-60 and TRA-1-81, and alkaline phosphatase (AP) enzymatic activities in P4-iPSCs. Note that a mouse pluripotency marker SSEA1 is negative. (**d**) The normal karyotype of P4-iPSCs (46, XY [20]). (**e**) The global gene expression patterns were compared between P4-iPSCs and P4-fibroblasts, and between P4-iPSCs and human ESCs (H9) using microarray analyses. (**f**) A bisulfite sequencing analysis of the *OCT4* and *NANOG* promoters in P4-fibroblasts and P4-iPSCs. (**g**) After 16 days of differentiation with embryoid body (EB) formation, cells were stained with anti-βIII-tubulin (ectoderm), SMA (mesoderm) and SOX17 (endoderm) antibodies. (**h**) Teratomas were generated by injecting P4-iPSCs into the testes of immunocompromised NOD-SCID mice. Neural tissue (ectoderm; left panel), cartilage tissue (mesoderm; right, arrowheads) and intestinal epithelia (endoderm; right, arrows) were observed. Scale bars, 100 μm. See also Figures S1–S6, Tables 1, S1 and S2.

We obtained skin samples from seven ADPKD patients (P1–7) whose characteristics are shown in Table 1. All the patients met the clinical diagnostic criteria for ADPKD^29^. Four (P1, P3, P4 and P7) out of the seven patients had ICAs, while the remaining three patients did not. P1, P3 and P4 were diagnosed as having ICAs by magnetic resonance angiography (MRA) within three years before the skin biopsy for iPSC derivation and P7 was diagnosed as having ICAs 26 years before the biopsy by angiography. P4 also had a past history of subarachnoid hemorrhage (SAH). All three patients without ICAs (P2, P5 and P6) were examined by MRA within three years before and after the skin biopsy for P2 and P5 and six years before and five years after the biopsy for P6. All patients except P6 had hypertension, and P3 was diagnosed as having temporal arteritis at the time of the biopsy. The skin fibroblasts were converted into iPSCs after transduction either with four retroviral vectors encoding OCT4, SOX2, KLF4 and c-MYC or with three vectors encoding OCT4, SOX2 and KLF4 (Fig. 1a,b)^30^,^31^. The different combinations of factors does not cause molecular or cellular differences in the resulting iPSCs^31^. Quantitative PCR with reverse transcription (qRT-PCR) was used to evaluate ADPKD-iPSC clones with repression of the exogenously introduced genes (Figure S1). Using these analyses, one iPSC clone whose repression level was highest for each of the seven patients was selected for further examination in order to minimize the effects of the exogenously introduced genes on the differentiated cells (Table S1). The genetic identity between patient fibroblasts and the corresponding iPSCs was confirmed by short tandem repeat (STR) analyses (Table S2).

All seven iPSC clones exhibited characteristic human embryonic stem cell (hESC) morphology (Fig. 1b) and were positively stained with pluripotency markers, including OCT4, NANOG, SOX2, SSEA4, TRA-1–60, TRA-1–81, and alkaline phosphatase (AP), but not a mouse ESC/iPSC marker, SSEA1 (Figs 1c and S2). These clones also exhibited mRNA expression of multiple pluripotency markers, including *NANOG*, *GDF3*, *DPPA4*, *TERT* and *REX1* (Figure S3), and normal karyotypes, except for minimal abnormality in one clone, P3-iPSC (Fig. 1d and Table S1). Microarray analysis showed that the global gene expression profiles of ADPKD-iPSCs were more similar to those of hESCs (H9) than to those of the parental fibroblasts (Figs 1e and S4). We observed an obvious decrease in the methylation level of the *OCT4* and *NANOG* promoters in ADPKD-iPSCs compared with the parental fibroblasts using bisulfite sequencing analysis (Figs 1f and S5). ADPKD-iPSCs could be differentiated into all three germ layers by embryoid body (EB) and teratoma formation following the intratesticular injection of undifferentiated ADPKD-iPSCs into non-obese diabetic/severe combined immunodeficient (NOD-SCID) mice (Fig. 1g,h). These results suggest that ADPKD-iPSCs have similar pluripotent properties to previously reported human ESC/iPSC lines^30^,^32^.

The mutational analyses of three patients (P4, P6 and P7) and their families were performed with multipoint linkage analyses, which demonstrated that all three had a mutation in the *PKD1* gene (Table 1 and Figure S6)^33^. The genomic analysis showed that the ADPKD-fibroblasts and -iPSCs possessed both wild-type and mutant *PKD1* alleles in all three patients (Figure S6). We examined the mutation status of the iPSC lines and the parental fibroblasts from the remaining four patients (P1, P2, P3 and P5) by exome analyses and long-range PCR with next-generation sequencing^34^. The whole sequences of the *PKD1*/*PKD2* genes except for exons 8–12 of the *PKD1* gene were determined, and the genomic mutations that resulted in amino acid changes were identified in both iPSC lines and the original fibroblasts from three (P2, P3 and P5) out of the four patients (Table 1). We could not identify the genetic mutation of P1 in the exons of *PKD1*/*PKD2* genes, but found intronic mutations 8161 + 38G > A and 2986-15C > T, which are described in ADPKD Mutation Database [PKDB] (http://pkdb.mayo.edu/) (Table 1).

### Differentiation of ADPKD-iPSCs into Vascular Cells

**Figure 2:**
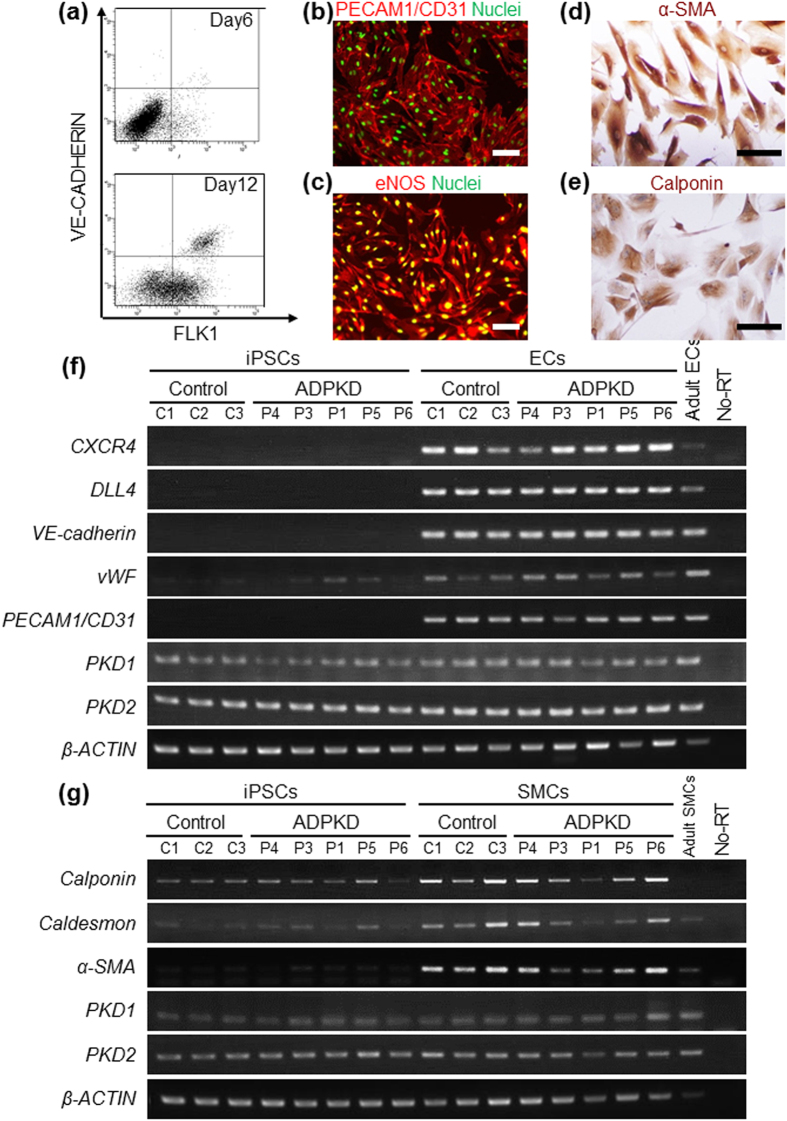
Directed Differentiation of ADPKD-iPSCs into Vascular Endothelia and Smooth Muscle Cells. (**a**) Flow cytometric analyses of ADPKD iPSC-derived cells on culture days 6 (upper) and 12 (lower). (**b**,**c**) Immunostaining of vascular endothelial cells (ECs) on day 12 differentiated from P4-iPSC-derived VE-CADHERIN (+) FLK1 (+) cells for the endothelial markers PECAM1/CD31 (**b**) and endothelial NO synthase (eNOS) (**c**). (**d**,**e**) Immunostaining of vascular smooth muscle cells (SMCs) on day 25 differentiated from P4-iPSC-derived VE-CADHERIN (−) FLK1 (+) cells for the smooth muscle cell markers α-smooth muscle actin (αSMA) (**d**) and Calponin (**e**). (**f**) The expression of marker genes (*CXCR4*, *DLL4*, *VE-cadherin*, *vWF* and *PECAM1/CD31*) and *PKD1* and *PKD2* in ECs derived from three control-iPSC (C1-3) and five ADPKD-iPSC lines (P4, P3, P1, P5 and P6). (**g**) The expression of marker genes (*Calponin*, *Caldesmon* and *α-SMA*) and *PKD1* and *PKD2* in SMCs derived from the control- and ADPKD-iPSC lines. Scale bars, 100 μm. See also Figures S7–S9.

We used eleven iPSC clones (C1–11) established from eight non-ADPKD Japanese subjects as control-iPSCs (Table S3)^35^. To create cellular disease models for vascular lesions, ADPKD- and control-iPSCs were differentiated into vascular endothelia and smooth muscle cells from sorted FLK1 (+) VE-cadherin (+) and FLK1 (+) VE-cadherin (−) cells on culture day 8, respectively, using our previously reported differentiation protocols (Fig. 2a)^36^,^37^,^38^. Vascular endothelia and smooth muscle cells differentiated from ADPKD-iPSCs showed similar vascular marker expression to previously reported human iPSC-derived vascular cells (Figs 2b–g and S7). These results indicate that ADPKD-iPSCs can be differentiated into vascular endothelia and smooth muscle cells. Both the vascular endothelia and smooth muscle cells derived from ADPKD- and control-iPSCs showed similar morphologies (Figure S9a) and no significant differences in the expression levels of vascular marker genes, *PKD1* and *PKD2* (Figure S8). Then, we compared the proliferation and angiogenic potentials between the endothelia derived from ADPKD- and control-iPSCs using immunostaining with a proliferation marker Ki67 and tube formation assay, respectively, finding no significant differences in either assay (Figure S9b–d)^37^.

### Altered Calcium Handling in Vascular Cells Differentiated from ADPKD-iPSCs

**Figure 3:**
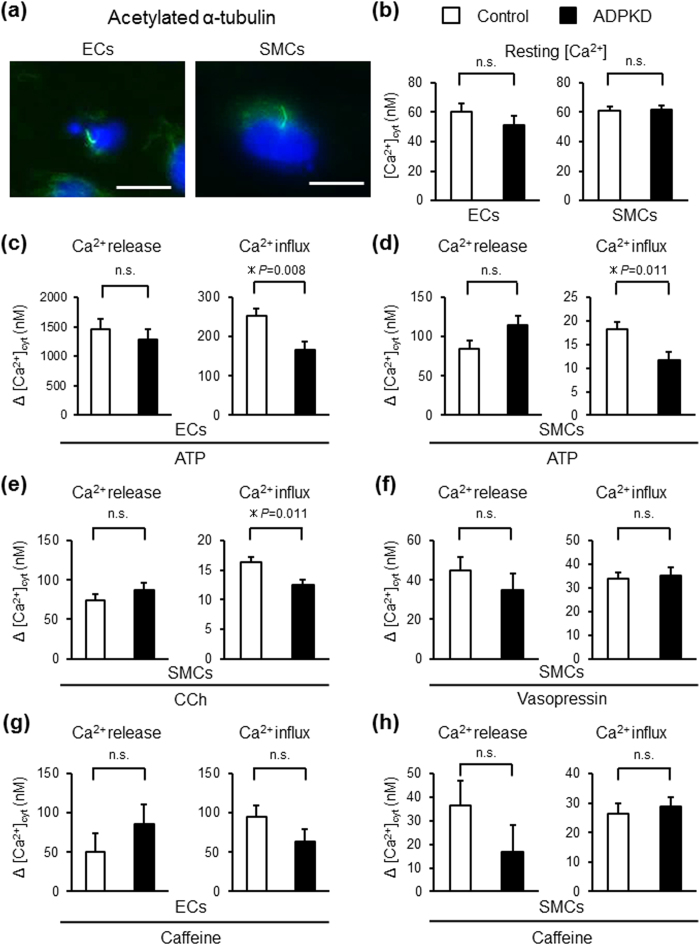
Intracellular Ca^2+^ Regulation in Vascular Endothelia and Smooth Muscle Cells Derived from Control- and ADPKD-iPSCs. (**a**) Anti-acetylated α-tubulin immunostaining of vascular endothelia (ECs) on culture day 12 and smooth muscle cells (SMCs) on day 25 differentiated from P4-iPSCs shows primary cilia (green). (**b**) The resting cytosolic Ca^2+^ concentration of ECs and SMCs derived from control-iPSCs (white bars) and ADPKD-iPSCs (black bars). (**c**–**h**) ATP (**c**,**d**), carbachol (CCh) (**e**), vasopressin (**f**) and caffeine (**g**,**h**) induced Ca^2+^ release and influx in ECs (**c**,**g**) and SMCs (**d**–**f**,**h**) derived from control- and ADPKD-iPSCs. Ten control- and seven ADPKD-iPSC lines and eight to ten control- and seven ADPKD-iPSC lines were used for the analyses of ECs (**b**,**c**,**g**) and SMCs (**b**,**d**–**f**,**h**), respectively. The results are expressed as the means ± SEM. *Statistically significant, n.s.; not significant. See also Tables S4 and S5.

Defects in either polycystin-1 or polycystin-2 expressed in primary cilia are thought to disrupt the intracellular Ca^2+^ regulation, eventually leading to renal cyst formation or vascular lesions associated with ADPKD^10^. Abnormal intracellular Ca^2+^ handling was detected in vascular smooth muscle cells from *Pkd1*^+/−^ and *Pkd2*^+/−^ mouse aortas, renal collecting duct cells from *Pkd1*^+/−^ mice and renal cyst cells from ADPKD patients^24^,^25^,^27^,^39^,^40^,^41^. We first confirmed the presence of primary cilia in iPSC-derived vascular endothelia and smooth muscle cells with immunostaining for acetylated α-tubulin (Fig. 3a).

Then, in order to examine whether the vascular cells from ADPKD-iPSCs showed altered intracellular Ca^2+^ regulation, we measured the resting [Ca^2+^]_i_, agonist-induced Ca^2+^ release from the intracellular stores and Ca^2+^ entry using the fluorescent probe fura-2. While almost all iPSC-derived endothelia responded to ATP stimulation, only 12~38% of cells responded to carbachol (CCh) stimulation (Tables S4 and S5). We therefore used ATP as the agonist for the inositol 1,4,5-triphosphate (IP3)-IP3 receptor pathway in endothelia in subsequent analyses. In contrast, more than 50% of the iPSC-derived vascular smooth muscle cells responded to ATP or CCh stimulation, while 6–8% of cells responded to vasopressin. In addition, a small percentage of iPSC-derived endothelia or smooth muscle cells responded to caffeine stimulus. The percentage of cells responding to each of the four agonists was not significantly different between the vascular cells derived from ADPKD- and control-iPSCs (Tables S4 and S5).

The resting [Ca^2+^]_i_ levels and Ca^2+^ release from the intracellular stores induced by the four agonists were not significantly different between the vascular cells derived from ADPKD- and control-iPSCs (Fig. 3b–h and Table S5). In contrast, the Ca^2+^ entry under stimulation with ATP or CCh, but not vasopressin or caffeine, showed a statistically significant decrease in both endothelia and smooth muscle cells derived from ADPKD-iPSCs compared with those derived from control-iPSCs (Fig. 3c–h, and Table S5). These differences were statistically significant even after considering the effects of age, sex, original cell types from which iPSCs were generated and iPSC derivation methods (*P* = 0.008 for endothelia, *P* = 0.013 and 0.036 for smooth muscle cells induced by ATP and CCh, respectively). However, no significant differences in these parameters for intracellular Ca^2+^ handling were observed between vascular cells from ADPKD patients with ICAs (P1, P3, P4 and P7) and those from patients without ICAs (P2, P5 and P6; Table S5). These results suggest that the vascular cells derived from ADPKD-iPSCs show an altered intracellular Ca^2+^ handling.

### Identification of Disease-related Molecules using iPSC-derived Vascular Cells

**Figure 4:**
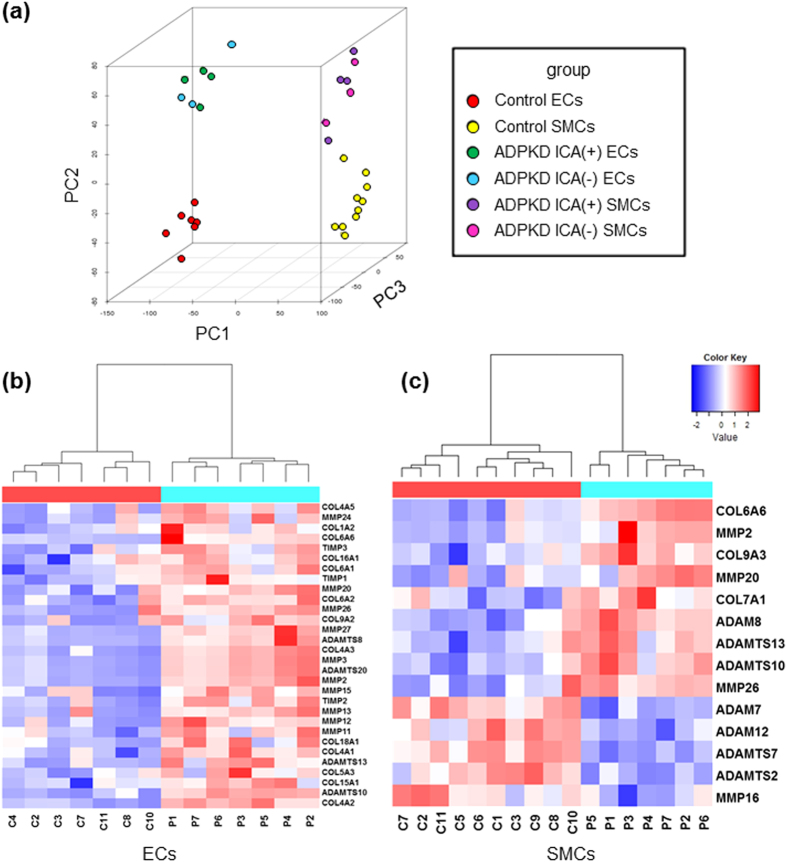
Altered Gene Expression Profiles of Vascular Endothelia and Smooth Muscle Cells Differentiated from ADPKD-iPSCs. (**a**) The gene expression profiles of vascular endothelia (ECs) and smooth muscle cells (SMCs) derived from ADPKD- and control-iPSCs were analyzed using a principal component analysis (PCA). The first, second and third principal components’ contribution ratios were 0.404, 0.103 and 0.036, respectively. (**b**,**c**) The expression levels of multiple genes responsible for extracellular matrix metabolism were altered in ECs (**b**) and SMCs (**c**) derived from ADPKD-iPSCs compared with those derived from control-iPSCs. Genes with *P*-values less than 0.05 were listed and heat maps were generated. The z-scores were calculated from the average and standard deviation of all scores. High and low scores are indicated by red and blue, respectively. Microarray analysis was performed with one sample for each cell line in (**a**–**c**). PC, principal component; ICA, intracranial aneurysm.

In an attempt to identify candidate molecules that might be involved in the pathogenesis of the vasculopathy in ADPKD, the expression profiles of vascular cells derived from ADPKD- and control-iPSCs were examined by microarray analyses. A principal component analysis (PCA) three-dimensional plot showed that there were differential gene expression patterns for the vascular cells derived from ADPKD- and control-iPSCs (Fig. 4a). Because the abnormal expression of extracellular matrix metabolism-related genes was reported in previous studies with rat and zebrafish PKD models, we focused on these genes in the analysis of vascular cells differentiated from ADPKD-iPSCs^42^,^43^. Consistently, we found that the expression levels of multiple genes involved in extracellular matrix metabolism were significantly altered in vascular cells derived from ADPKD-iPSCs compared to those from control-iPSCs (Fig. 4b,c).

Vascular cells derived from the ADPKD patient group with ICAs (P1, P3, P4 and P7) and the patient group without ICAs (P2, P5 and P6) were compared in order to identify molecules that might be associated with ICA formation in ADPKD. However, there were no candidate genes whose expression levels were significantly altered between the two groups (adjusted *P*-value <0.05; t-test with Holm multiple test correction). Accordingly, we compared each of the four ADPKD patients with ICAs with each of the three patients without ICAs for each gene and selected candidates that had an average fold change in expression of more than two. We thus found 17 and 5 candidates that had their expression upregulated in iPSC-derived endothelia and smooth muscle cells from the ADPKD patients with ICAs compared to those from patients without ICAs (Tables S6 and S7). In contrast, no genes were consistently downregulated.

**Figure 5:**
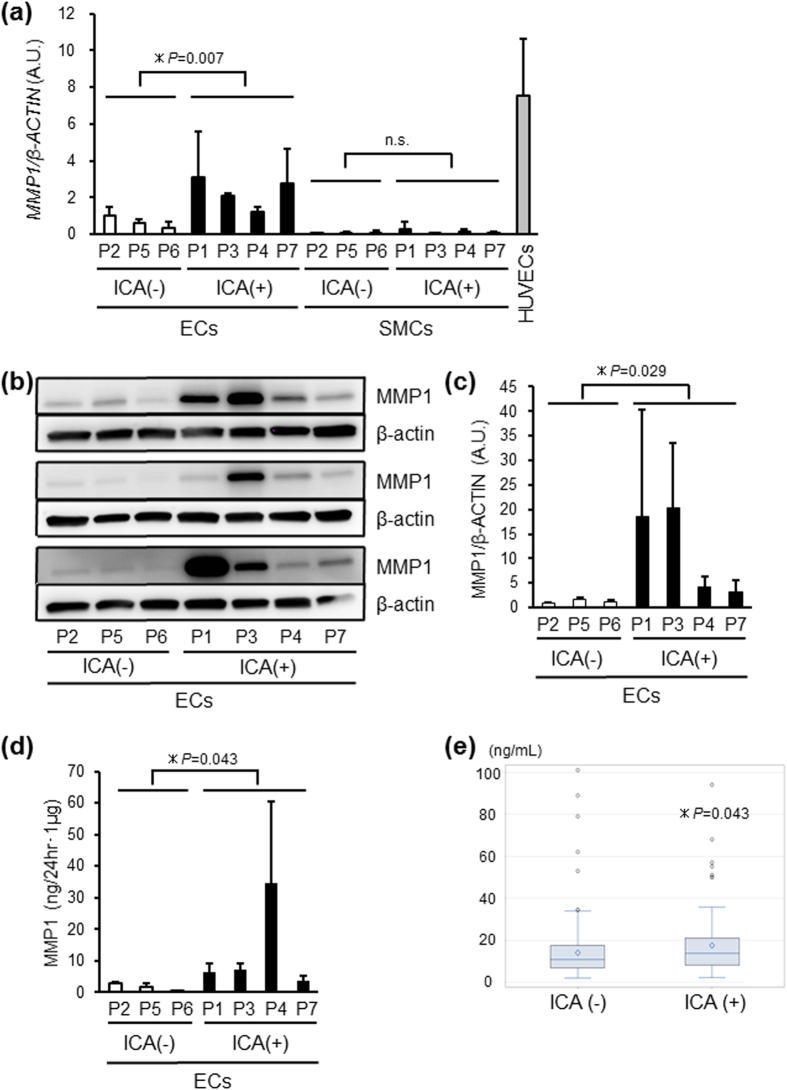
MMP1 Expression is Specifically Upregulated in iPSC-derived Endothelia from ADPKD Patients with ICA. (**a**) The expression levels of *MMP1* in iPSC-derived endothelial cells (ECs) from the four ADPKD patients with intracranial aneurysms (ICAs; P1, P3, P4 and P7) were significantly higher than those from the three patients without ICAs (P2, P5 and P6). Each value was normalized to the average of the iPSC-derived EC samples from P2. (**b**,**c**) There was significant upregulation of MMP1 protein expression in iPSC-derived ECs from the four ADPKD patients with ICAs compared to those from the three patients without ICAs, as confirmed by a Western blotting analysis of total cell lysates. Each value was normalized to the average of the iPSC-derived EC samples from P2. (**d**) The secretion of the MMP1 protein into the culture media from iPSC-derived ECs from the four ADPKD patients with ICAs was significantly higher than those from the three patients without ICAs as assessed by ELISAs. The secreted MMP1 protein level was normalized to the total cellular protein weight of each cell lysate sample from iPSC-derived endothelial cell cultures. (**e**) A comparison of the serum MMP1 levels from the 97 ADPKD patients with ICAs and the 257 patients without ICAs. The data from three (**a**,**c**) and five (**d**) independent experiments are presented as the means ± SD. SMCs; smooth muscle cells, HUVECs; human umbilical vein endothelial cells, *statistically significant, n.s.; not significant. See also Figures S10, S11, Tables 2 and S6–S9.

Next, we sought to identify a serum marker specific for ADPKD patients with ICAs, and analyzed the protein sequence of the candidates. We selected seven (*MMP10*, *MMP1*, *PCSK1*, *BMP6*, *EDN1*, *CTGF* and *TFPI2*) and two (*MMP1* and *TFPI2*) out of the 17 and 5 candidates for endothelia and smooth muscle cells, respectively, that encoded secreted proteins (where secreted proteins were defined as having a signal peptide sequence and no or one transmembrane region). qRT-PCR analyses of the vascular cells from all seven ADPKD-iPSCs were performed to examine the reproducibility of the microarray data and showed that only the *MMP1* expression was significantly upregulated in the iPSC-derived endothelia from the four ADPKD patients with ICAs compared with the three patients without ICAs (*P* = 0.007; Figs 5a and S10). In contrast, the expression level of *MMP1* in smooth muscle cells was very low, as evaluated by the qRT-PCR analyses (Fig. 5a). Volcano plots drawn with fold changes and uncorrected *P*-values of microarray data showed that MMP1 was remarkably plotted (Figure S11). We also confirmed that both the expression and secretion of the MMP1 protein, as analyzed by Western blotting analysis and enzyme-linked immunosorbent assay (ELISA), respectively, were significantly higher in endothelia from ADPKD-iPSCs derived from patients with ICAs compared with those from patients without ICAs (*P* = 0.029 for protein expression and *P* = 0.043 for secretion; Fig. 5b–d).

We then evaluated the diagnostic accuracy of MMP1 assays to predict the presence of ICAs in ADPKD patients. Serum samples from 97 ADPKD patients with ICAs (cases) and 257 patients without ICAs (controls) were collected and analyzed for their MMP1 levels using an ELISA (Table S8). All patients undergo MRA examinations every three years for those without ICAs and every year for those with ICAs. The results showed that ADPKD patients with ICAs had higher MMP1 serum concentrations than those without (mean ± SD; 17.6 ± 14.9 vs 14.0 ± 12.1 ng/mL; *P* = 0.043; Fig. 5e). Using receiver operating characteristic (ROC) analyses to determine the highest sensitivity and specificity values that could be obtained, we set the serum MMP1 level to 15 ng/mL as the cutoff for ADPKD patients to indicate the likelihood of having ICAs (odds ratio (95% CI); 2.031 (1.260–3.274); *P* = 0.0036; Tables 2 and S9). The sensitivity and specificity of MMP1 were low at 0.48 and 0.68, respectively. However, in a multivariate analysis where the patients’ age, sex, family history of ICAs or subarachnoid hemorrhage (SAH), renal function (serum creatinine level), dialysis therapy and MMP1 levels were taken into account as potential confounding factors, the serum MMP1 level was confirmed to be one of the risk factors associated with ICAs formation in ADPKD patients (adjusted odds ratio (95% CI); 1.891 (1.139–3.138); *P* = 0.014; Table 2). The addition of serum MMP1 levels to the multivariate logistic regression analysis increased the values of the area under a ROC curve (AUC) from 0.653 to 0.672 and decreased the value of Akaike’s information criterion (AIC) from 403.1 to 399.0 (Table 2). Family history of ICAs or SAH, which is reported to be risk factors for the complication of ICAs in ADPKD, sex (female), which is a known to be a risk factor for ICAs in the generalized population, and serum MMP1 level were significantly associated with the complication of ICAs in ADPKD in our clinical study (Table 2)^44^,^45^. These results suggest that the vascular cells derived from ADPKD-iPSCs may be used to identify novel disease-related molecules.

## Discussion

Although ICAs are one of the most serious cardiovascular complications in ADPKD, little is known about the pathogenesis of these vascular lesions, and novel cellular assay systems to elucidate the disease mechanisms are needed. In this study, we demonstrated that the vascular cells differentiated from patient-derived iPSCs could be used to identify molecules associated with the vasculopathy of ADPKD.

Vascular cells derived from ADPKD-iPSCs showed altered Ca^2+^ entry under stimulation with ATP or CCh, but not with vasopressin or caffeine. One possible explanation is that the receptors or downstream signaling molecules for these agonists were not sufficiently expressed in vascular cells derived from iPSCs. This is consistent with the finding of this study that a small percentage of cells responded to the vasopressin or caffeine stimulus. Although the difference of Ca^2+^ entry in iPSC-derived smooth muscle cells was small between controls and ADPKD patients, it has been reported that an altered local Ca^2+^ increase just below the channels can exert significant physiological effects on cell behavior^46^,^47^, which suggests that the small differences observed between smooth muscle cells derived from control- and ADPKD-iPSCs may lead to different phenotypes. While vascular smooth muscle cells from *Pkd1*^+/−^ and *Pkd2*^+/−^ mouse aortas showed a statistically significant decrease in the resting [Ca^2+^]_i_ levels and agonist-induced Ca^2+^ release in previous studies^24^,^25^,^27^,^39^,^40^,^41^, we found human vascular cells derived from ADPKD-specific iPSCs did not. One possible explanation for the inconsistency is the species difference in the correlation between the gene expression levels of *PKD1* or *PKD2* and the phenotypic presentation. Other possibilities include the functional differences between vascular cells differentiated from iPSCs and primary cultured vascular cells. Nevertheless, the present findings suggest that vascular cells derived from ADPKD-specific iPSCs can recapitulate one aspect of *in vivo* disease phenotypes.

The expression levels of multiple genes involved in extracellular matrix metabolism were significantly altered in vascular cells derived from ADPKD-iPSCs compared to those derived from control-iPSCs, as shown by the microarray analyses. These data are in line with the results of expression profiling studies of the kidney from rat and zebrafish PKD models^42^,^43^. Other reports have also described the abnormal expression of matrix metalloproteinase genes, including MMP9 in cell lines from the liver cysts of ADPKD patients^48^, MMP2 and MMP14 in kidneys of PKD murine and rat models^49^,^50^,^51^, and higher serum MMP1 and MMP9 levels in ADPKD patients^52^.

In this study, the mRNA and protein expression and protein secretion levels of MMP1 for each ADPKD-iPSC line were not consistent and had large variation. One possible reason for the inconsistency and variation is that the samples were collected in different experiments because of the limited number of cells obtained in one experiment. Nevertheless, MMP1 was consistently and significantly upregulated in the iPSC-derived endothelia from ADPKD patients with ICAs compared with the patients without ICAs in multiple assays. On the other hand, the expression level of MMP1 in iPSC-derived smooth muscle cells was very low, suggesting that the upregulated MMP1 expression by endothelia is the main associate of ICA formation.

Despite the altered expression of multiple genes involved in extracellular matrix metabolism, a differential expression of MMP1 was not observed between the endothelia derived from ADPKD- and control-iPSCs, but was observed between iPSC-derived endothelia from ADPKD patients with ICAs and those from ADPKD patients without ICAs. An increased expression of matrix metalloproteinase genes, especially MMP2 and MMP9, has been reported in aneurysmal tissues, which occurred in the absence of ADPKD, compared to normal arteries^53^,^54^,^55^,^56^. Extending these findings to our results indicates that MMP1 may be associated with the pathogenesis of ICA formation in ADPKD. It is possible that the elevation of MMP1 may reflect inflammatory conditions at the site of the aneurysms. Another possibility is that the systemic elevation of MMP1 may reflect generalized endothelial cell abnormalities which increase the vulnerability of the vascular wall to mechanical stresses from the blood flow in the circle of Willis, thus eventually leading to the formation of ICAs. Our findings could also potentially be more broadly applied to aneurysm formation in the non-ADPKD general population.

Although intra-familial variability in ADPKD is much lower than inter-familial variability and some genotype-phenotype correlations have been reported in the development of ICAs in ADPKD patients, the finding that not all ADPKD individuals of high-risk families develop ICAs indicates that other genetic variants may be involved besides the *PKD1* or *PKD2* mutations^14^,^15^,^45^. In our study, vascular cells from ADPKD-iPSCs showed impaired intracellular Ca^2+^ homeostasis, which may result from a *PKD1* or *PKD2* mutation, compared with those from control-iPSCs, but no significant differences were observed between vascular cells from ADPKD patients with and without ICAs, in spite of the differences in the expression of some genes, including *MMP1*. These results suggest that other genetic modifiers besides *PKD1* and *PKD2* are associated with the development of ICAs in ADPKD. MMP1 may be a disease-related molecule involved in the pathogenesis of ICA formation in ADPKD, and its expression is likely regulated by such genetic modifiers.

In our clinical study, we examined the serum MMP1 levels of 354 ADPKD patients, and found that ADPKD patients with ICAs had higher MMP1 serum concentrations than those without ICAs although the difference was small. One possible explanation for the small difference is that the non-ICA group may include patients who will develop ICAs in the future because our study was cross-sectional. We examined multiple clinical parameters, such as age, sex, family history of ICAs or subarachnoid hemorrhage (SAH), serum creatinine level and the presence or absence of dialysis therapy, in addition to the serum MMP1 levels (Tables 2, S8 and S9). We found the serum MMP1 level is the second strongest parameter among the 6 factors examined by univariate analysis. It is stronger than family history of ICAs or SAH and age, which are reported to be risk factors for the complication of ICAs in ADPKD, and sex, which is a known risk factor for ICAs in the general population^44^,^45^. Although univariate analysis found the serum creatinine level was stronger than the serum MMP1 level, multivariate analysis of all 6 factors found the serum creatinine level not significant (Table 2). Considering that we examined 5 common clinical parameters, our analysis shows that the serum MMP1 level is one risk factor for the development of ICAs in ADPKD patients. These results support organizing a prospective cohort study in the future to properly evaluate the diagnostic performance of serum MMP1 levels for predicting the development or current presence of ICAs in ADPKD patients. Additionally, a low-cost serum test would help determine which patients should undergo a MRA examination, which is currently used as a primary diagnostic method for complicated ICAs in ADPKD patients.

We could not identify the genetic mutation of P1 in the exons of *PKD1*/*PKD2*, but intronic mutations 8161 + 38G > A and 2986-15C > T were found in P1, which are described in ADPKD Mutation Database [PKDB] (http://pkdb.mayo.edu/.mutation). In the *PKD1*/*PKD2* mutation analysis, we could not specifically amplify the sequences of exons 8–12 of the *PKD1* gene from the genomic DNAs because of the presence of six highly homologous sequences. Thus, one possible explanation for not identifying the exonic mutations of P1 is that the mutations may be in the exons 8–12 of the *PKD1* gene. On the other hand, two recent papers showed that some ADPKD patients in Japan and China do not harbor patient-specific mutations in the exonic regions of either *PKD1* or *PKD2*^57^,^58^. Although we cannot exclude the possibility of the mutations in the *PKD1* or *PKD2* genes of P1, there may be other causative mutations in ADPKD patients including P1. There is also a possibility that intronic mutations deposited in ADPKD Mutation Database might behave as causal mutations in ADPKD pathogenesis. Future investigation using next-generation whole genome sequencing analysis with long read techniques, which have the advantage of sequencing repetitive sequences, should provide reliable genotyping of the *PKD* genes and may disclose other candidate causative genes.

One of the advantages of disease modeling research using patient-derived iPSCs is that it makes it possible to address the symptomatic variability of human diseases, whereas mouse disease models generated by genetic manipulation show relatively uniform disease phenotypes, which do not reflect the variability of disease phenotypes among patients. The characteristic features of ADPKD include the intra- and inter-familial symptomatic variability, and a typical example is complicated ICAs, the prevalence of which was reported to be around 8%^1^. We generated iPSCs from ADPKD patients with and without ICAs, and identified MMP1 as a possible molecule associated with this variability in the complication of ICAs among the ADPKD patients. iPSC models reduce the importance of age in comparative studies, as the telomere length of iPSCs is independent of the subject’s age^59^. In fact, we found in the present study larger differences in the gene expression patterns between vascular cells derived from ADPKD- and control-iPSCs than those among control-iPSC lines derived from subjects with a wide range of age. These results suggest that the impact of age is less than that of the genetic background. Future studies to create an *in vitro* model for aneurysm formation using vascular cells derived from ADPKD-iPSCs would lead to a better understanding of the mechanisms of aneurysm formation in ADPKD patients. These approaches using patient-derived iPSCs can also be applied to other disorders to elucidate the mechanisms underlying the symptomatic variability among patients.

In conclusion, the vascular cells derived from ADPKD-specific iPSCs can be used as a powerful tool for elucidating the mechanisms underlying the development of vascular complications in ADPKD. More broadly, our results suggest the usefulness of cellular disease models developed using patient-specific iPSCs for identifying novel disease-related molecules that may be used in clinical practice.

## Materials and Methods

### Ethics Statement

This study was approved by the Ethics Committees of Kyoto University and Toranomon Hospital and conducted according to the guidelines of the Declaration of Helsinki. All patients provided written informed consent. Animal experiments were approved by the CiRA Animal Experiment Committee, and conducted in accordance with the institutional guidelines.

### *In Vitro* Differentiation into Vascular Cells

The differentiation of iPSCs into vascular cells was carried out as described previously^36^,^37^. Briefly, undifferentiated human iPSCs were harvested and transferred to a collagen I-coated dish after adjusting the colonies to an appropriate size. On the second day of incubation, the culture medium was replaced with Primate ES medium (ReproCELL) without basic fibroblast growth factor (bFGF), supplemented with N2 supplement (Thermo Fisher Scientific)/B27 supplement (Thermo Fisher Scientific) and 6-bromoindirubin-3′-oxime (BIO, 5 μM, SIGMA). Thereafter, the cells were incubated for another 3 days, at which time the culture medium was replaced with StemPro-34 SFM (Thermo Fisher Scientific) supplemented with recombinant human vascular endothelial growth factor (VEGF, 50 ng/ml, PeproTech). After another 4–8 days of incubation, FLK1 (+) VE-cadherin (+) and FLK1 (+) VE-cadherin (−) cells were sorted individually using a FACSAria flow cytometer (BD) and were used for the subsequent experiments. Sorted FLK1 (+) VE-cadherin (+) cells were confirmed to remain VE-cadherin positive during the subsequent cell culture and analyses. On the other hand, the sorted FLK1 (+) VE-cadherin (−) cells were differentiated into vascular smooth muscle cells after an additional 18–22 days of differentiation on collagen I-coated dishes supplemented with recombinant human platelet-derived growth factor-BB (PDGF-BB, 20 ng/ml, PeproTech). Anti-FLK1 monoclonal antibody was kindly provided by Kyowa Hakko Kirin Co., Ltd., Tokyo, Japan and used at 1:200. Anti-VE-cadherin monoclonal antibody (560410, BD Pharmingen) was used at 1:8.

### Measurement of Changes in the [Ca^2+^]i

Fura-2 fluorescence was measured in HEPES-buffered saline (HBS) containing 107 mM NaCl, 6 mM KCl, 1.2 mM MgSO_4_, 2 mM CaCl_2_, 11.5 mM glucose, and 20 mM HEPES, adjusted to pH 7.4 with NaOH. The fluorescent images were analyzed with a video image analysis system (AQUACOSMOS, Hamamatsu Photonics). Ratio images of 340:380 were obtained on a pixel-by-pixel basis. Fura-2 measurements were carried out at 25 ± 1 °C in HBS adjusted to pH 7.4. The 340:380 ratio images were converted to Ca^2+^ concentrations by *in vivo* calibration using 5 μM ionomycin, as described previously^60^. To measure Ca^2+^ release from the intracellular stores, the cells were incubated with Ca^2+^-free solution containing 0.5 mM EGTA without CaCl_2_ before Ca^2+^ release was evoked by adding 100 μM ATP, 100 μM CCh, 10 ng/ml vasopressin or 10 mM caffeine in Ca^2+^-free solution. The Ca^2+^ entry was measured after the re-addition of Ca^2+^ into the solution.

### Microarray Analysis

Total RNA from skin fibroblasts, human iPSCs (hiPSCs), hESCs and vascular cells derived from hiPSCs was isolated using the RNeasy Mini Kit (Qiagen). One hundred nanograms of total RNA from each sample was processed using the WT Expression Kit (Ambion) and the WT Terminal Labeling Kit (Affymetrix). Samples were prepared for hybridization by labeling cDNA in a 1X hybridization cocktail according to the manufacturer’s instructions. GeneChip arrays (Human Gene 1.0 ST) were hybridized in a GeneChip Hybridization Oven at 45 °C for 16 h at 60 RPM. Washing was done with a GeneChip Fluidics Station 450, according to the manufacturer’s instructions, with the buffers provided in the Affymetrix GeneChip Hybridization, Wash, and Stain Kit. The arrays were scanned on a GeneChip Scanner 3000 7G plus (Affymetrix), and raw data processing was performed using AGCC software (version 2.0, Affymetrix). Raw data in the form of CEL files were imported into the GeneSpring GX (version 11.0) software program (Agilent Technologies). The normalization and probe summarization were performed by the Robust multi-array average (RMA) method. The prediction of the signal peptide sequence was performed using the Signal P 4.1 Server (http://www.cbs.dtu.dk/services/SignalP/). Transmembrane regions were predicted by the SOSUI algorithm (http://bp.nuap.nagoya-u.ac.jp/sosui/).

The gene expression profiling of vascular cells derived from ADPKD- and control-iPSCs was performed using the SurePrint G3 Human Gene Expression 8 × 60K v2 Microarray Kit (Agilent Technologies). The data were normalized using GeneSpring GX 12.6.1 software as follows: (i) threshold raw signals were set to 1.0, (ii) log base 2 transformation was performed, and (iii) 75th percentile normalization was chosen. Genes differently expressed between two conditions were listed using a multiple statistical test controlled by a false discovery rate (FDR) at 0.05 by Tukey’s range test. The microarray data are available at Gene Expression Omnibus (GEO; http://www.ncbi.nlm.nih.gov/geo) with series accession number GSE74453.

### Serum Specimen Collection from ADPKD Patients

A total of 354 ADPKD patients were analyzed for their serum MMP1 levels. All patients were Japanese. The presence of intracranial aneurysms (ICAs) was tested by magnetic resonance angiography (MRA) in all the patients. The profiles of the patients are shown in Table S8.

### Testing for MMP1 Levels

MMP1 concentrations in the serum samples were measured by SRL. Inc., Japan using a commercially available enzyme-linked immunosorbent assay (Daiichi Fine Chemical CO., LTD, Japan). The serum samples obtained were stored at −80 °C until the analysis.

### Statistical Methods

For the baseline variables, summary statistics were constructed employing frequencies and proportions for categorical data, and the means, standard deviations and ranges for continuous variables. The comparability of the baseline characteristics between the two groups was assessed by means of a two-sample *t*-test for continuous variables or by using Fisher’s exact test for categorical variables. For the repeated measurement data in the Ca^2+^ experiments, after a variance stabilizing transformation, a linear mixed model was applied to test for differences between cases and controls over time, and a compound symmetry covariance structure was used to model the data. A multiple logistic regression analysis was performed to examine the effects of MMP1, age, sex, family history, dialysis and renal function on the incidence of cerebral aneurysms. Adjusted odds ratios (OR) and their 95% confidence intervals (CI) were estimated by the model.

All comparisons were planned, and the tests were two-sided. A *P*-value <0.05 was considered to indicate a statistically significant difference. For the multiple testing problem, we applied the Holm method on the computed *P*-values to reduce the risk of type I errors. All statistical analyses were conducted using SAS software, version 9.4 (SAS Institute Inc., Cary, NC, USA).

## Additional Information

**How to cite this article**: Ameku, T. *et al*. Identification of MMP1 as a novel risk factor for intracranial aneurysms in ADPKD using iPSC models. *Sci. Rep.* **6**, 30013; doi: 10.1038/srep30013 (2016).

## Supplementary Material

Supplementary Information

## Figures and Tables

**Table 1:** Profiles of seven ADPKD patients whose dermal fibroblasts were converted into iPSCs.

Patient	Age at skin biopsy	Sex	Clinical features	Molecular Defect (in exon)	Vascular complications	eGFR (ml/min per 1.73 m^2^)	Dialysis
P1	45Y	Female	Numerous kidney cysts; Family history	ND*	HT, ICA	<15	+
P2	64Y	Female	Numerous kidney cysts; No family history	W429S, S2235L, V3008M, S3404Y, S3405Y, Missense, *PKD1* gene	HT	54	−
P3	71Y	Male	Numerous kidney cysts; No family history	A190T, Missense, *PKD2* gene	HT, ICA, temporal arteritis	<15	+
P4	49Y	Male	Numerous kidney cysts; Family history	G3818R, Missense, *PKD1* gene	HT, ICA, SAH	10	−
P5	41Y	Male	Numerous kidney cysts; Family history	E2111K, R2327W, Missense, *PKD1* gene	HT	65	−
P6	45Y	Female	Numerous kidney cysts; Family history	Q3895X, Nonsense, *PKD1* gene	−	43	−
P7	69Y	Female	Numerous kidney cysts; Family history	7024delAC, Frameshift, *PKD1* gene	HT, ICA	<15	+

ND*, not determined in the Exons. Intronic mutations 8161 + 38G > A and 2986-15C > T were found, which are described in ADPKD Mutation Database [PKDB]. (http://pkdb.mayo.edu/). HT, hypertension; ICA, intracranial aneurysm; SAH, subarachnoid hemorrhage.

**Table 2:** Multiple logistic regression analysis of MMP1, age, sex, family history, dialysis and renal function as risk factors for the incidence of intracranial aneurysms.

Risk factors	Multivariate analysis		
	Adjusted odd ratio	95% CI	*P*-value
(a) Model without serum MMP1 levels			
Age ≧65Y	1.505	0.853–2.657	0.16
Sex (female)	1.809	1.086–3.013	0.023
Family history of ICAs or SAH	2.089	1.207–3.615	0.0084
Creatinine ≧2 mg/dL	1.133	0.533–2.410	0.75
Dialysis therapy	1.987	0.893–4.419	0.092
(b) Model including serum MMP1 levels			
MMP1 ≧15 ng/mL	1.891	1.139–3.138	0.014
Age ≧65Y	1.537	0.864–2.735	0.14
Sex (female)	1.851	1.104–3.103	0.020
Family history of ICAs or SAH	2.165	1.243–3.768	0.0063
Creatinine ≧2 mg/dL	1.018	0.475–2.185	0.96
Dialysis therapy	1.918	0.857–4.290	0.11

(**a**) AUC: 0.653 (95%CI: 0.589–0.716), AIC: 403.11. (**b**) AUC: 0.672 (95%CI: 0.609–0.734), AIC: 399.04. ICA, intracranial aneurysm; SAH, subarachnoid hemorrhage; AUC, area under the curve; AIC, Akaike’s information criterion.
