# Supplementary material for: Identification of MMP1 as a novel risk factor for intracranial aneurysms in ADPKD using iPSC models
Source: Sci Rep. 2016 Jul 15;6:30013. doi: 10.1038/srep30013 (PMC4945931; doi:10.1038/srep30013)
Supplement: Supplementary Information [file srep30013-s1.pdf]

**SUPPLEMENTARY INFORMATION**

# Identification of MMP1 as a novel risk factor for intracranial aneurysms in ADPKD using iPSC models

Tomonaga Ameku, Daisuke Taura, Masakatsu Sone, Tomohiro Numata, Masahiro Nakamura, Fumihiko Shiota, Taro Toyoda, Satoshi Matsui, Toshikazu Araoka, Tetsuhiko Yasuno, Shin-Ichi Mae, Hatasu Kobayashi, Naoya Kondo, Fumiyo Kitaoka, Naoki Amano, Sayaka Arai, Tomoko Ichisaka, Norio Matsuura, Sumiko Inoue, Takuya Yamamoto, Kazutoshi Takahashi, Isao Asaka, Yasuhiro Yamada, Yoshifumi Ubara, Eri Muso, Atsushi Fukatsu, Akira Watanabe, Yasunori Sato, Tatsutoshi Nakahata, Yasuo Mori, Akio Koizumi, Kazuwa Nakao, Shinya Yamanaka and Kenji Osafune

**Table S1. Characterization of established iPSC clones, Related to Figure 1.**

| Patient | Clone number | Factors used | Marker expression | | Pluripotency | | Karyotype | STR | BS |
|---|---|---|---|---|---|---|---|---|---|
| | | | RT-PCR | IC | EB | Teratoma | | | |
| P1 | CiRA00004 | 3F | ✔ | ✔ | ✔ | ✔ | 46,XX[20] | ✔ | ✔ |
| P2 | CiRA00005 | 3F | ✔ | ✔ | ✔ | ✔ | 46,XX[20] | ✔ | ✔ |
| P3 | CiRA00006 | 4F | ✔ | ✔ | ✔ | ✔ | 48,XY,+X,+12[2] /46,XY[18] | ✔ | ✔ |
| P4 | CiRA00007 | 4F | ✔ | ✔ | ✔ | ✔ | 46,XY[20] | ✔ | ✔ |
| P5 | CiRA00008 | 4F | ✔ | ✔ | ✔ | ✔ | 46,XY[20] | ✔ | ✔ |
| P6 | CiRA00009 | 4F | ✔ | ✔ | ✔ | ND | 46,XX[20] | ✔ | ✔ |
| P7 | CiRA00010 | 4F | ✔ | ✔ | ✔ | ✔ | 46,XX[20] | ✔ | ✔ |

IC, immunocytochemistry; EB, embryoid body; STR, short tandem repeat analysis; BS, bisulfite sequencing of OCT4 and NANOG promoters; 3F, iPSCs generated with three reprogramming factors, OCT4, SOX2 and KLF4; 4F, iPSCs generated with four factors, OCT4, SOX2, KLF4 and c-MYC; ND, not done.

**Table S2. STR analyses of ADPKD-iPSCs and their parental fibroblasts, Related to Figure 1.**

| Locus/Cells | P1-fibroblast | | P1-iPSC | | P2-fibroblast | | P2-iPSC | | P3-fibroblast | | P3-iPSC | | P4-fibroblast | | P4-iPSC | | P5-fibroblast | | P5-iPSC | |
|---|---|---|---|---|---|---|---|---|---|---|---|---|---|---|---|---|---|---|---|---|
| D3S1358 | 16 | 17 | 16 | 17 | 15 | 16 | 15 | 16 | 15 | 17 | 15 | 17 | 16 | 17 | 16 | 17 | 15 | 16 | 15 | 16 |
| TH01 | 6 | | 6 | | 9 | | 9 | | 7 | 9 | 7 | 9 | 7 | 9 | 7 | 9 | 7 | | 7 | |
| D21S11 | 29 | 33.2 | 29 | 33.2 | 29 | 31 | 29 | 31 | 29 | 30 | 29 | 30 | 29 | | 29 | | 31 | 31.2 | 31 | 31.2 |
| D18S51 | 13 | 22 | 13 | 22 | 14 | 16 | 14 | 16 | 18 | 19 | 18 | 19 | 12 | 13 | 12 | 13 | 17 | 21 | 17 | 21 |
| Penta_E | 19 | 21 | 19 | 21 | 12 | 16 | 12 | 16 | 19 | 20 | 19 | 20 | 9 | 19 | 9 | 19 | 15 | 21 | 15 | 21 |
| D5S818 | 12 | 13 | 12 | 13 | 11 | 12 | 11 | 12 | 9 | 13 | 9 | 13 | 10 | | 10 | | 7 | 12 | 7 | 12 |
| D13S317 | 8 | | 8 | | 8 | 11 | 8 | 11 | 9 | 11 | 9 | 11 | 10 | 11 | 10 | 11 | 11 | 12 | 11 | 12 |
| D7S820 | 12 | | 12 | | 10 | 12 | 10 | 12 | 9 | 13 | 9 | 13 | 8 | | 8 | | 8 | 14 | 8 | 14 |
| D16S539 | 12 | 13 | 12 | 13 | 9 | 12 | 9 | 12 | 12 | 14 | 12 | 14 | 9 | 12 | 9 | 12 | 9 | 10 | 9 | 10 |
| CSF1PO | 12 | | 12 | | 7 | 9 | 7 | 9 | 10 | | 10 | | 12 | | 12 | | 9 | 11 | 9 | 11 |
| Penta_D | 9 | 12 | 9 | 12 | 11 | | 11 | | 9 | 14 | 9 | 14 | 9 | 11 | 9 | 11 | 9 | | 9 | |
| AMEL | X | | X | | X | | X | | X | Y | X | Y | X | Y | X | Y | X | Y | X | Y |
| vWA | 14 | | 14 | | 16 | 18 | 16 | 18 | 16 | 17 | 16 | 17 | 18 | | 18 | | 16 | 17 | 16 | 17 |
| D8S1179 | 11 | 15 | 11 | 15 | 11 | 16 | 11 | 16 | 12 | 15 | 12 | 15 | 14 | 17 | 14 | 17 | 12 | 14 | 12 | 14 |
| TPOX | 9 | 10 | 9 | 10 | 8 | 11 | 8 | 11 | 8 | 12 | 8 | 12 | 8 | 10 | 8 | 10 | 8 | 11 | 8 | 11 |
| FGA | 21 | 22 | 21 | 22 | 20 | 22 | 20 | 22 | 23 | 24 | 23 | 24 | 23 | 24 | 23 | 24 | 22 | | 22 | |

| Locus/Cells | P6-fibroblast | | P6-iPSC | | P7-fibroblast | | P7-iPSC | |
|---|---|---|---|---|---|---|---|---|
| D3S1358 | 15 | 17 | 15 | 17 | 15 | 16 | 15 | 16 |
| TH01 | 7 | 9 | 7 | 9 | 7 | 9 | 7 | 9 |
| D21S11 | 30 | 30.2 | 30 | 30.2 | 30 | 32.2 | 30 | 32.2 |
| D18S51 | 15 | 17 | 15 | 17 | 14 | 15 | 14 | 15 |
| Penta_E | 5 | 15 | 5 | 15 | 11 | 15 | 11 | 15 |
| D5S818 | 10 | 12 | 10 | 12 | 10 | 11 | 10 | 11 |
| D13S317 | 11 | 12 | 11 | 12 | 11 | | 11 | |
| D7S820 | 8 | 11 | 8 | 11 | 12 | | 12 | |
| D16S539 | 10 | 11 | 10 | 11 | 12 | 13 | 12 | 13 |
| CSF1PO | 11 | | 11 | | 9 | 12 | 9 | 12 |
| Penta_D | 9 | 11 | 9 | 11 | 9 | 12 | 9 | 12 |
| AMEL | X | | X | | X | | X | |
| vWA | 14 | 17 | 14 | 17 | 17 | 18 | 17 | 18 |
| D8S1179 | 10 | 15 | 10 | 15 | 10 | | 10 | |
| TPOX | 9 | 11 | 9 | 11 | 8 | 9 | 8 | 9 |
| FGA | 20 | 22 | 20 | 22 | 20 | 25 | 20 | 25 |

**Table S3. Profiles of the eleven control iPSC lines.**

| Clones | | Age | Sex | Original Cells | Derivation Method | Reference |
|---|---|---|---|---|---|---|
| C1 | TIG107 4F1 | 81Y | Female | Fibroblasts | Retrovirus OSKM | Tomoda et al., 2012; Koyanagi-Aoi et al., 2013 |
| C2 | TIG114 4F1 | 36Y | Male | Fibroblasts | Retrovirus OSKM | Tomoda et al., 2012; Koyanagi-Aoi et al., 2013 |
| C3 | TIG118 4F1 | 12Y | Female | Fibroblasts | Retrovirus OSKM | Tomoda et al., 2012; Koyanagi-Aoi et al., 2013 |
| C4 | TIG119 4F1 | 6Y | Male | Fibroblasts | Retrovirus OSKM | |
| C5 | TIG120 4F1 | 6Y | Female | Fibroblasts | Retrovirus OSKM | Tomoda et al., 2012; Koyanagi-Aoi et al., 2013 |
| C6 | TIG120 3F7 | 6Y | Female | Fibroblasts | Retrovirus OSK | Tomoda et al., 2012; Koyanagi-Aoi et al., 2013 |
| C7 | TIG121 4F4 | 6M | Male | Fibroblasts | Retrovirus OSKM | |
| C8 | TIG975E4 | 6Y | Female | Fibroblasts | Episomal OSK, L-MYC, LIN28, and shRNA for TP53 | |
| C9 | TIG975E2 | 6Y | Female | Fibroblasts | Episomal OSK, L-MYC, LIN28, and shRNA for TP53 | |
| C10 | 585B1 | 30s | Male | T cells | Episomal OSK, L-MYC, LIN28, and shRNA for TP53 | Okita et al.,2013 |
| C11 | TIG107 3F1 | 81Y | Female | Fibroblasts | Retrovirus OSK | Tomoda et al., 2012; Koyanagi-Aoi et al., 2013 |

O, OCT4; S, SOX2; K, KLF4; M, C-MYC.

**Table S4. The percentage of cells responding to each agonist, Related to Figures 3 and S10.**

| | Endothelial cells | | | Smooth muscle cells | | |
|---|---|---|---|---|---|---|
| Agonist | ADPKD | Control | *P*-value | ADPKD | Control | *P*-value |
| ATP | 97.2±2.0 | 99.3±2.0 | 0.45 | 52.5±9.7 | 59.2±8.5 | 0.61 |
| CCh | ND | ND | ND | 53.1±8.5 | 51.3±7.9 | 0.88 |
| Vasopressin | ND | ND | ND | 6.3±1.2 | 7.8±NA | 0.24 |
| Caffeine | 2.9±9.1 | 13.2±7.6 | 0.40 | 2.6±1.3 | 5.1±1.3 | 0.20 |

The results are expressed as the means ± SEM. CCh, carbachol; ND, not done; NA, not applicable.

**Table S5. The result of $Ca^{2+}$ experiments using iPSC-derived vascular cells, Related to Figures 3 and S10.**

(**a**) iPSC-derived endothelia

| | Resting $[Ca^{2+}]_i$ (nM) | ATP | | | | Caffeine | | | |
|---|---|---|---|---|---|---|---|---|---|
| | | Release $\Delta[Ca^{2+}]_i$ (nM) | Influx $\Delta[Ca^{2+}]_i$ (nM) | Resp (%) | Number of analyzed cells (Number of experiments) | Release $\Delta[Ca^{2+}]_i$ (nM) | Influx $\Delta[Ca^{2+}]_i$ (nM) | Resp (%) | Number of analyzed cells (Number of experiments) |
| P1 | 52.4 | 1253.8 | 204.3 | 100.0 | 419(5) | 67.5 | 52.7 | 2.5 | 394(5) |
| P2 | 41.4 | 1139.4 | 159.9 | 100.0 | 157(2) | 0.0 | 34.8 | 0.0 | 171(2) |
| P3 | 42.1 | 2426.6 | 185.3 | 100.0 | 188(2) | 160.2 | 46.4 | 1.5 | 200(2) |
| P4 | 50.0 | 1199.8 | 177.8 | 100.0 | 225(3) | 48.8 | 92.6 | 2.8 | 258(3) |
| P5 | 78.1 | 898.8 | 130.6 | 84.2 | 300(5) | 136.7 | 94.9 | 7.5 | 200(3) |
| P6 | 27.0 | 1262.0 | 177.7 | 99.0 | 100(1) | 0.0 | 78.8 | 0.0 | 100(1) |
| P7 | 57.8 | 761.6 | 103.2 | 100.0 | 100(1) | 228.7 | 44.3 | 6.0 | 100(1) |
| C1 | 32.3 | 1485.6 | 468.7 | 100.0 | 100(2) | 288.3 | 174.2 | 100.0 | 100(2) |
| C2 | 48.0 | 902.0 | 161.3 | 99.0 | 100(1) | 0.0 | 152.1 | 0.0 | 100(1) |
| C3 | 66.6 | 1646.4 | 289.2 | 100.0 | 188(4) | 0.0 | 87.8 | 0.0 | 182(2) |
| C4 | 85.8 | 1330.1 | 192.9 | 98.8 | 337(4) | 26.1 | 65.2 | 2.7 | 337(4) |
| C5 | 83.2 | 1006.1 | 219.4 | 100.0 | 100(3) | 105.5 | 50.7 | 21.0 | 100(1) |
| C6 | 54.7 | 1270.3 | 212.8 | 100.0 | 100(1) | 0.0 | 41.9 | 0.0 | 100(1) |
| C7 | 64.9 | 2751.3 | 276.6 | 100.0 | 87(1) | 33.6 | 239.8 | 2.0 | 100(1) |
| C8 | 58.1 | 2234.9 | 277.6 | 98.7 | 177(2) | 28.7 | 39.5 | 0.7 | 171(2) |
| C9 | 39.8 | 1077.0 | 253.7 | 99.0 | 300(3) | 80.0 | 109.8 | 5.7 | 300(3) |
| C10 | 56.5 | 990.7 | 203.8 | 100.0 | 100(1) | 0.0 | 61.3 | 0.0 | 82(1) |

(**b**) iPSC-derived smooth muscle cells

| | Resting $[Ca^{2+}]cyt$ (nM) | ATP | | | | CCh | | | |
|---|---|---|---|---|---|---|---|---|---|
| | | Release $\Delta[Ca^{2+}]_i$ (nM) | Influx $\Delta[Ca^{2+}]_i$ (nM) | Resp (%) | Number of analyzed cells (Number of experiments) | Release $\Delta[Ca^{2+}]_i$ (nM) | Influx $\Delta[Ca^{2+}]_i$ (nM) | Resp (%) | Number of analyzed cells (Number of experiments) |
| P1 | 56.3 | 106.8 | 10.3 | 67.0 | 96(1) | 91.4 | 10.2 | 45.0 | 95(1) |
| P2 | 63.6 | 157.6 | 4.4 | 53.1 | 100(2) | 79.7 | 11.9 | 64.0 | 75(1) |
| P3 | 56.7 | 117.9 | 14.2 | 78.0 | 100(1) | 67.3 | 14.9 | 47.0 | 87(1) |
| P4 | 80.2 | 123.6 | 18.1 | 59.5 | 82(1) | 120.9 | 15.4 | 87.0 | 89(1) |
| P5 | 55.9 | 135.5 | 10.7 | 92.0 | 100(2) | 101.5 | 10.3 | 91.0 | 100(1) |
| P6 | 62.9 | 113.7 | 10.8 | 5.0 | 100(1) | 71.8 | 12.6 | 21.0 | 100(1) |
| P7 | 63.3 | 44.5 | 13.0 | 12.5 | 88(1) | 76.1 | 12.6 | 17.0 | 98(1) |
| C1 | 64.5 | 105.2 | 16.8 | 78.5 | 100(2) | 57.3 | 14.0 | 37.0 | 91(2) |
| C2 | 53.5 | 110.3 | 18.9 | 55.0 | 62(2) | 119.3 | 14.2 | 60.0 | 96(2) |
| C3 | 50.8 | 63.6 | 18.1 | 73.0 | 100(1) | 71.3 | 22.5 | 71.0 | 100(1) |
| C4 | 78.8 | 102.4 | 11.7 | 69.0 | 36(1) | | | | |
| C5 | 54.5 | 64.3 | 16.3 | 49.4 | 100(1) | 65.8 | 16.8 | 43.0 | 89(3) |
| C6 | 54.4 | | | | | | | | |
| C7 | 58.0 | 79.6 | 15.7 | 56.0 | 100(2) | 88.1 | 16.6 | 62.0 | 100(4) |
| C8 | 61.3 | 58.8 | 23.1 | 47.0 | 100(1) | 58.8 | 15.7 | 55.0 | 100(1) |
| C9 | 55.2 | 45.9 | 15.9 | 22.0 | 100(1) | 33.1 | 14.3 | 28.0 | 200(2) |
| C10 | 83.7 | | | | | 99.8 | 16.6 | 55.0 | 94(1) |
| C11 | 69.3 | 127.9 | 27.9 | 83.0 | 100(1) | | | | |

| | Vasopressin | | | | Caffeine | | | |
|---|---|---|---|---|---|---|---|---|
| | Release $\Delta[Ca^{2+}]_i$ (nM) | Influx $\Delta[Ca^{2+}]_i$ (nM) | Resp (%) | Number of analyzed cells (Number of experiments) | Release $\Delta[Ca^{2+}]_i$ (nM) | Influx $\Delta[Ca^{2+}]_i$ (nM) | Resp (%) | Number of analyzed cells (Number of experiments) |
| P1 | 44.1 | 39.3 | 4.7 | 438(5) | 6.9 | 29.8 | 2.4 | 478(5) |
| P2 | 21.9 | 27.3 | 7.4 | 353(4) | 13.7 | 14.7 | 3.3 | 350(4) |
| P3 | 39.7 | 40.1 | 8.2 | 462(5) | 23.6 | 27.1 | 2.8 | 500(6) |
| P4 | 0.0 | 26.5 | 0.0 | 36(1) | 13.9 | 23.8 | 1.0 | 36(1) |
| P5 | 41.1 | 32.4 | 15.7 | 290(4) | 35.8 | 35.7 | 6.5 | 200(3) |
| P6 | 33.3 | 42.3 | 3.7 | 486(5) | 13.0 | 35.3 | 1.9 | 583(6) |
| P7 | 40.4 | 26.3 | 3.2 | 210(3) | 10.4 | 35.0 | 0.4 | 204(3) |
| C1 | 102.2 | 43.7 | 16.7 | 180(3) | 7.8 | 24.1 | 3.0 | 114(2) |
| C2 | 53.5 | 25.0 | 7.9 | 339(4) | 63.0 | 18.3 | 2.8 | 78(2) |
| C3 | 50.8 | 23.7 | 7.8 | 664(8) | 62.7 | 29.9 | 5.0 | 248(4) |
| C4 | 6.0 | 41.4 | 11.7 | 310(4) | 0.0 | 40.5 | 0.0 | 56(2) |
| C5 | 43.2 | 31.2 | 4.7 | 384(8) | 3.5 | 19.5 | 1.1 | 237(4) |
| C6 | 37.3 | 43.5 | 3.1 | 200(2) | 27.6 | 23.8 | 11.0 | 200(2) |
| C7 | 72.1 | 45.3 | 11.1 | 252(6) | 22.0 | 17.3 | 15.2 | 79(2) |
| C8 | 22.3 | 39.8 | 8.7 | 394(4) | 15.5 | 47.4 | 1.1 | 178(2) |
| C9 | 32.4 | 26.9 | 3.9 | 385(4) | 139.8 | 16.0 | 4.7 | 92(1) |
| C10 | 0.0 | 19.8 | 0.0 | 54(1) | | | | |
| C11 | | | | | | | | |

Resp (%); the percentage of cells responding to each agonist.

**Table S6. The results of the microarray analyses comparing the gene expression profiles of iPSC-derived vascular endothelia from ADPKD patients with intracranial aneurysms and those from ADPKD patients without ICAs, Related to Figure 5.**

The gene expression profiles of iPSC-derived vascular endothelia from four ADPKD patients with ICAs (P1, P3, P4 and P7) were individually compared with those from three patients without ICAs (P2, P5 and P6). From these 12 comparisons, genes with an average fold change in expression of more than two were selected.

(**a**) List of 17 candidate genes

| Gene Symbol | Description | RefSeq | Probe ID | Localization | | Gene chip score | | | | | | | uncorrected *P*-value (t-test) |
|---|---|---|---|---|---|---|---|---|---|---|---|---|---|
| | | | | Signal peptide | 14 | P1 | P2 | P3 | P4 | P5 | P6 | P7 | |
| SPOCD1 | SPOC domain containing 1 | NM_144569.4 | 7914467 | No | 0 | 537.5 | 360.5 | 541.9 | 806.3 | 265.6 | 335.3 | 1546.5 | 9.78E-03 |
| PLXNA2 | Plexin A2 | NM_025179.3 | 7923991 | Yes | 3 | 1800.6 | 1703.1 | 2112.7 | 2299.3 | 579.0 | 1122.0 | 2756.8 | 1.50E-02 |
| MYEOV | Myeloma overexpressed gene (in a subset of t(11;14) positive multiple myelomas) | NM_138768.2 | 7942118 | No | 3 | 153.4 | 152.1 | 160.3 | 327.9 | 148.4 | 126.2 | 629.0 | 9.95E-02 |
| MMP10 | Matrix metallopeptidase 10 (stromelysin 2) | NM_002425.2 | 7951259 | Yes | 1 | 197.6 | 120.3 | 135.1 | 140.6 | 65.9 | 78.7 | 1042.8 | 6.36E-02 |
| MMP1 | Matrix metallopeptidase 1 (interstitial collagenase) | NM_002421.3 | 7951271 | Yes | 0 | 6827.3 | 1050.7 | 4954.3 | 4125.5 | 1487.5 | 506.8 | 12831.2 | 5.27E-04 |
| E2F7 | E2F transcription factor 7 | NM_203394.2 | 7965094 | No | 0 | 519.6 | 463.1 | 791.4 | 1132.5 | 431.7 | 348.9 | 1716.8 | 1.49E-02 |
| SLC30A3 | Solute carrier family 30 (zinc transporter), member 3 | NM_003459.4 | 8051050 | No | 5 | 362.9 | 220.6 | 490.6 | 630.4 | 215.3 | 239.0 | 524.0 | 4.25E-04 |
| SNAI1 | Snail homolog 1 (Drosophila) | NM_005985.3 | 8063382 | No | 0 | 939.7 | 223.1 | 463.3 | 792.3 | 279.9 | 314.0 | 352.6 | 1.32E-02 |
| NR2F1 | Nuclear receptor subfamily 2, | NM_005654.4 | 8106923 | No | 0 | 930.3 | 427.0 | 933.4 | 1066.8 | 553.2 | 614.7 | 1258.0 | 5.99E-04 |

| | | | | | | | | | | | | | |
|---|---|---|---|---|---|---|---|---|---|---|---|---|---|
| | group F, member 1 | | | | | | | | | | | | |
| PCSK1 | Proprotein convertase subtilisin/kexin type 1 | NM_000439.4 | 8113234 | Yes | 0 | 1629.9 | 569.6 | 691.7 | 1169.1 | 670.8 | 370.1 | 2188.3 | 1.21E-02 |
| BMP6 | Bone morphogenetic protein 6 | NM_001718.4 | 8116818 | Yes | 0 | 3127.2 | 1772.9 | 2943.1 | 3535.6 | 1475.8 | 1403.5 | 3026.6 | 6.07E-05 |
| EDN1 | Endothelin 1 | NM_001955.4 | 8116921 | Yes | 0 | 7147.1 | 4570.0 | 9345.0 | 8857.1 | 3981.2 | 2327.3 | 9656.2 | 8.33E-04 |
| CTGF | Connective tissue growth factor | NM_001901.2 | 8129562 | Yes | 0 | 10662.0 | 7513.3 | 8142.4 | 9285.0 | 2361.1 | 3284.2 | 11640.2 | 7.81E-03 |
| TFPI2 | Tissue factor pathway inhibitor 2 | NM_006528.3 | 8141016 | Yes | 0 | 9092.9 | 2006.5 | 2415.7 | 3074.4 | 1457.4 | 1849.8 | 3198.4 | 3.44E-02 |
| NRCAM | Neuronal cell adhesion molecule | NM_001037132.2 | 8142270 | Yes | 2 | 551.3 | 139.1 | 285.1 | 310.7 | 54.8 | 90.7 | 1129.2 | 2.10E-03 |
| CD274 | CD274 molecule | NM_014143.3 | 8154233 | Yes | 2 | 228.3 | 111.0 | 135.3 | 122.0 | 53.8 | 77.8 | 517.2 | 2.59E-02 |
| FRMD3 | FERM domain containing 3 | NM_174938.5 | 8161964 | No | 1 | 128.2 | 127.2 | 223.6 | 367.1 | 93.3 | 117.7 | 258.0 | 1.66E-02 |

(**b**) Fold change in the 12 comparisons.

| | SPOCD1 | | | PLXNA2 | | | MYEOV | | | MMP10 | | | MMP1 | | | E2F7 | | |
|---|---|---|---|---|---|---|---|---|---|---|---|---|---|---|---|---|---|---|
| | P2 | P5 | P6 | P2 | P5 | P6 | P2 | P5 | P6 | P2 | P5 | P6 | P2 | P5 | P6 | P2 | P5 | P6 |
| P1 | 1.49 | 2.02 | 1.60 | 1.06 | 3.11 | 1.60 | 1.01 | 1.03 | 1.22 | 1.64 | 3.00 | 2.51 | 6.50 | 4.59 | 13.5 | 1.12 | 1.20 | 1.49 |
| P3 | 1.50 | 2.04 | 1.62 | 1.24 | 3.65 | 1.88 | 1.05 | 1.08 | 1.27 | 1.12 | 2.05 | 1.72 | 4.72 | 3.33 | 9.78 | 1.71 | 1.83 | 2.27 |
| P4 | 2.24 | 3.04 | 2.40 | 1.35 | 3.97 | 2.05 | 2.16 | 2.21 | 2.60 | 1.17 | 2.13 | 1.79 | 3.93 | 2.77 | 8.14 | 2.45 | 2.62 | 3.25 |
| P7 | 4.29 | 5.82 | 4.61 | 1.62 | 4.76 | 2.46 | 4.14 | 4.24 | 4.98 | 8.66 | 15.8 | 13.2 | 12.2 | 8.63 | 25.3 | 3.71 | 3.98 | 4.92 |
| | SLC30A3 | | | SNAI1 | | | NR2F1 | | | PCSK1 | | | BMP6 | | | EDN1 | | |
| | P2 | P5 | P6 | P2 | P5 | P6 | P2 | P5 | P6 | P2 | P5 | P6 | P2 | P5 | P6 | P2 | P5 | P6 |
| P1 | 1.65 | 1.69 | 1.52 | 4.21 | 3.36 | 2.99 | 2.18 | 1.68 | 1.51 | 2.86 | 2.43 | 4.40 | 1.76 | 2.12 | 2.23 | 1.56 | 1.79 | 3.07 |
| P3 | 2.22 | 2.28 | 2.05 | 2.08 | 1.66 | 1.48 | 2.19 | 1.69 | 1.52 | 1.21 | 1.03 | 1.87 | 1.66 | 1.99 | 2.10 | 2.04 | 2.35 | 4.02 |
| P4 | 2.86 | 2.93 | 2.64 | 3.55 | 2.83 | 2.52 | 2.50 | 1.93 | 1.74 | 2.05 | 1.74 | 3.16 | 1.99 | 2.40 | 2.52 | 1.94 | 2.22 | 3.81 |
| P7 | 2.38 | 2.43 | 2.19 | 1.58 | 1.26 | 1.12 | 2.95 | 2.27 | 2.05 | 3.84 | 3.26 | 5.91 | 1.71 | 2.05 | 2.16 | 2.11 | 2.43 | 4.15 |
| | CTGF | | | TFPI2 | | | NRCAM | | | CD274 | | | FRMD3 | | | | | |
| | P2 | P5 | P6 | P2 | P5 | P6 | P2 | P5 | P6 | P2 | P5 | P6 | P2 | P5 | P6 | | | |
| P1 | 1.42 | 4.52 | 3.25 | 4.53 | 6.24 | 4.92 | 3.96 | 10.1 | 6.08 | 2.06 | 4.25 | 2.94 | 1.01 | 1.37 | 1.09 | | | |
| P3 | 1.08 | 3.45 | 2.48 | 1.20 | 1.66 | 1.31 | 2.05 | 5.20 | 3.14 | 1.22 | 2.52 | 1.74 | 1.76 | 2.40 | 1.90 | | | |
| P4 | 1.24 | 3.93 | 2.83 | 1.53 | 2.11 | 1.66 | 2.23 | 5.67 | 3.43 | 1.10 | 2.27 | 1.57 | 2.89 | 3.93 | 3.12 | | | |
| P7 | 1.55 | 4.93 | 3.54 | 1.59 | 2.19 | 1.73 | 8.12 | 20.6 | 12.5 | 4.66 | 9.62 | 6.65 | 2.03 | 2.76 | 2.19 | | | |

**Table S7. The results of the microarray analyses comparing the gene expression profiles of iPSC-derived vascular smooth muscle cells from ADPKD patients with intracranial aneurysms and those from ADPKD patients without ICAs, Related to Figure 5.**

The gene expression profiles of the iPSC-derived vascular smooth muscle cells from four ADPKD patients with ICAs (P1, P3, P4 and P7) were individually compared with those from three patients without ICAs (P2, P5 and P6). From these 12 comparisons, genes with an average fold change in expression of more than two were selected.

(**a**) List of 5 candidate genes

| Gene Symbol | Description | RefSeq | Probe ID | Localization | | Gene chip score | | | | | | | uncorrected *P*-value (t-test) |
|---|---|---|---|---|---|---|---|---|---|---|---|---|---|
| | | | | Signal peptide | SOSUI | P1 | P2 | P3 | P4 | P5 | P6 | P7 | |
| MYPN | Myopalladin | NM_032578 | 7927827 | No | 0 | 78.9 | 58.3 | 231.8 | 67.5 | 50.2 | 54.5 | 101.5 | 5.98E-02 |
| MMP1 | Matrix metallopeptidase 1 (interstitial collagenase) | NM_002421 | 7951271 | Yes | 0 | 481.6 | 243.1 | 1209.4 | 565.0 | 137.3 | 126.2 | 556.5 | 1.32E-03 |
| HMGA2 | High mobility group AT-hook 2 | NM_000274 | 7956867 | No | 0 | 2892.3 | 916.3 | 2866.5 | 2468.6 | 1245.0 | 2167.0 | 2584.4 | 9.37E-03 |
| RPPH1 | Ribonuclease P RNA component H1 | NR_002312.1 | 7977507 | * | * | 2034.5 | 1223.7 | 3236.0 | 3408.7 | 954.7 | 1125.7 | 1804.0 | 2.56E-03 |
| TFPI2 | Tissue factor pathway inhibitor 2 | NM_006528 | 8141016 | Yes | 0 | 433.1 | 280.8 | 3923.8 | 1776.3 | 130.2 | 310.4 | 1706.2 | 8.28E-03 |

*: not available.

(**b**) Fold change in the 12 comparisons.

| | MYPN | | | MMP1 | | | HMGA2 | | | RPPH1 | | | TFPI2 | | |
|---|---|---|---|---|---|---|---|---|---|---|---|---|---|---|---|
| | P2 | P5 | P6 | P2 | P5 | P6 | P2 | P5 | P6 | P2 | P5 | P6 | P2 | P5 | P6 |
| P1 | 1.35 | 1.57 | 1.45 | 1.98 | 3.51 | 3.82 | 3.16 | 2.32 | 1.33 | 1.66 | 2.13 | 1.81 | 1.54 | 3.33 | 1.40 |
| P3 | 3.97 | 4.61 | 4.25 | 4.97 | 8.81 | 9.59 | 3.13 | 2.30 | 1.32 | 2.64 | 3.39 | 2.87 | 14.0 | 30.1 | 12.6 |
| P4 | 1.16 | 1.34 | 1.24 | 2.32 | 4.12 | 4.48 | 2.69 | 1.98 | 1.14 | 2.79 | 3.57 | 3.03 | 6.33 | 13.6 | 5.72 |
| P7 | 1.74 | 2.02 | 1.86 | 2.29 | 4.05 | 4.41 | 2.82 | 2.08 | 1.19 | 1.47 | 1.89 | 1.60 | 6.08 | 13.1 | 5.50 |

**Table S8. Profiles of ADPKD patients whose serum MMP1 levels were examined, Related to Figure 5.**

| | ICA (+) | ICA (-) |
|---|---|---|
| N | 97 | 257 |
| MMP1 (ng/mL), mean ± SD (range) | 17.6±14.9 (2.1-94) | 14.0±12.1 (1.8-101) |
| Age (Y), mean ± SD (range) | 58.8±10.6 (38-83) | 54.8±12.3 (22-81) |
| Male, n (%) | 31 (32) | 114 (44) |
| Family history of ICAs or SAH, n (%) | 32 (33) | 54 (21) |
| Creatinine (mg/dL), mean ± SD (range) | 4.92±3.5 (0.4-13.1) | 4.02±3.6 (0.5-15.9) |
| Dialysis therapy, n (%) | 54 (56) | 103 (40) |

ICA, intracranial aneurysm; SAH, subarachnoid hemorrhage.

**Table S9. Correlation between complicated intracranial aneurysms and serum MMP1 levels in ADPKD patients, Related to Figure 5.**

| | Univariate analysis | | | | |
|---|---|---|---|---|---|
| Risk factors | Odd ratio | 95% CI | *P*-value | AUC | AIC |
| MMP1≧15 ng/mL | 2.031 | 1.260-3.274 | 0.0036 | 0.584 | 410.67 |
| Age≧65Y | 1.799 | 1.072-3.020 | 0.026 | 0.557 | 414.27 |
| Sex (female) | 1.709 | 1.044-2.797 | 0.033 | 0.562 | 414.41 |
| Family history of ICAs or SAH | 1.842 | 1.096-3.095 | 0.021 | 0.559 | 413.91 |
| Creatinine≧2 mg/dL | 2.096 | 1.281-3.429 | 0.0032 | 0.588 | 410.05 |
| Dialysis therapy | 1.865 | 1.163-2.991 | 0.0096 | 0.577 | 412.34 |

ICA, intracranial aneurysm; SAH, subarachnoid hemorrhage; AUC, area under the curve; AIC, Akaike's information criterion.

**Table S10. Sequences of primers used in this study.**

| Gene | Forward primer; 5'-to-3' | Reverse primer; 5'-to-3' |
|---|---|---|
| hOCT4 Tg | GCTCTCCCATGCATTCAAACTGA | CCC TTT TTC TGG AGA CTA AAT AAA |
| hSOX2 Tg | TTCACATGTCCCAGCACTACCAGA | GACATGGCCTGCCCGGTTATTATT |
| hKLF4 Tg | CCACCTCGCCTTACACATGAAGA | GACATGGCCTGCCCGGTTATTATT |
| hcMYC Tg | ATACATCCTGTCCGTCCA AGCAGA | GACATGGCCTGCCCGGTTATTATT |
| hOCT4 Total | CCCCAGGGCCCCATTTTGGTACC | ACCTCAGTTTGAATGCATGGGAGAGC |
| hSOX2 Total | TTCACATGTCCCAGCACTACCAGA | TCACATGTGTGAGAGGGGCAGTGTGC |
| hKLF4 Total | GATTACGCGGGCTGCGGCAAAACCTACACA | TTAAAAATGTCTCTTCATGTGTAAGGCGAG |
| hcMYC Total | ATACATCCTGTCCGTCCAAGCAGA | TCACGCACAAGAGTTCCGTAGCTGTTCAAG |
| hPKD1 | AGGACTACGAGATGGTGGAGTT | CATCCCTTCAAAGCGGACTT |
| hPKD2 | GATTATGGAGCGAGCCAAAC | AGCGTTCCAACTCTTCACGTA |
| hMMP1 | CTGGGAGCAAACACATCTGA | AGTTCATGAGCTGCAACACG |
| hTFPI2 | GTCGATTCTGCTGCTTTTCC | CACTGGTCGTCCACACTCAC |
| hMMP10 | CCAGTCTGCTCTGCCTATCC | AACGTCAGGAACTCCACACC |
| hCTGF | ACTGTCCCGGAGACAATGAC | TGCTCCTAAAGCCACACCTT |
| hNRCAM | CCAGTCCATTCTGGGTCCTA | TGGCATGCTGTTGTATGCTT |
| hCD274 | GCCACCAGCTGTCATCACTA | CCAACACCACAAGGAGGAGT |

| | | |
|---|---|---|
| hSLC30A3 | TGCAGGTCGAGCAGTATCAG | GGTGGTAGGAGGGAGAGAGG |
| hNR2F1 | AGAAGCTCAAGGCGCTACAC | CCTACCAAACGGACGAAGAA |
| hBMP6 | TTCGTGCTGGAGTTTTGTTG | TGTGGCGTGGTATGCTGTAT |
| hEDN1 | CCATGAGAAACAGCGTCAAA | ATGGAAGCCAGTGAAGATGG |
| FRMD3 | GAGGGTGTTCCATTGCCTAA | CATAGCTCCACGACAAAGCA |
| hPLAXNA2 | TGCAAAGTGGCTTCACAGAC | GCTGTGATCGTGTCTCAGGA |
| hPCSK1 | CATGGCAACAGTTAGCTGGA | TGCGGGTAGTTTGTTTTTCC |
| hE2F7 | CGGGTTGGTTGTTCTAGCAT | GGAAGGAGGGAAGGAAGAGA |
| hSNAI1 | GGTTCTTCTGCGCTACTGCT | TAGGGCTGCTGGAAGGTAAA |
| hSPOCD | GCCGCCTGCTCTACTCATAC | AAGGAGAGGGGAGACAGGAA |
| hHMGA2 | AGCAGAAGCCACTGGAGAAA | GTGCCATATGGGGTAGCAGT |
| hRPPH1 | GTCACTCCACTCCCATGTCC | GGCGGAGGAGAGTAGTCTGA |
| hCXCR4 | GCCTTATCCTGCCTGGTATTGTC | GCGAAGAAAGCCAGGATGAGGAT |
| hDLL4 | GTGGACTGTGGCCTGGACAA | AGCATATCGCTGATATCCGACACTCTG |
| hVE-cadherin | ACACCTCACTTCCCCATCA | GACCTTGCCCACATATTCTCC |
| hvWF | GGGGTCATCTCTGGATTCAA | AGGCAAACATCTCCCACAAC |
| hPECAM1/CD31 | CAACGAGAAAATGTCAGA | GGAGCCTTCCGTTCTAGAGT |
| hCalponin | CTTCATGGACGGCCTCAAAGA | GTAGTTGTGTGCGTGGTGGTT |
| hCaldesmon | AGACAAGGAAAGAGCTGAGGCA | GCTGCTTGTTACGTTTCTGCTC |

| | | |
|---|---|---|
| hα-SMA | CACTGCCTTGGTGTGTGACAAT | CGTAGCTGTCTTTTTGTCCCATTC |
| hNANOG | CCTGAAGACGTGTGAAGATGAG | GCTGATTAGGCTCCAACCATAC |
| hDPPA4 | AGTGCCTGTTGCTTTGTGAGT | TGCACTGAACTGAGATTGCAC |
| hREX1 | AAAGCATCTCCTCATTCATGGT | TGGGCTTTCAGGTTATTTGACT |
| hGDF3 | CACCGTCACCAGCTATTCATTA | GTAGAGCATGGAAATGGGAGAC |
| hTERT | TGAAAGCCAAGAACGCAGGGATG | TGTCGAGTCAGCTTGAGCAGGAATG |
| β-ACTIN | CATGTACGTTGCTATCCAGGC | CTCCTTAATGTCACGCACGAT |
| PKD1(G3818R) | TAGTTCTCCAGGAGTGCCGC | CCGAGGTGAGCAGAGGCAG |
| PKD1(Q3895X) | AGGTGTGCCTGCTGCTGTT | AGTGAGGGCGTACAGCTGA |
| PKD1(G3818R), RFLP | TGCTGTTCGCCGTGCACTTCGCCGT | TAGTGAAGCGGCGCGGGC |
| PKD1(Q3895X), RFLP | TAGTTCTCCAGGAGTGCCGC | CCGAGGTGAGCAGAGGCAG |
| F26 | AGCGCAACTACTTGGAGGCCC | |
| R2LR | GCAGGGTGAGCAGGTGGGGCCATCCTAC | |
| PKDex15.14 | ATCATTGAGGGTGGCTCATAC | (6-Fam)TTCTCTGGGCTCATGGGTGT |
| PKD1 exon1 | CGCAGCCTTACCATCCACCT | TCATCGCCCCTTCCTAAGCA |
| PKD1 exon2-7 | CCCCGAGTAGCTGGAACTACAGTTACACACT | CGTCCTGCTGTGCCAGAGGCG |
| PKD1 exon8-12 | ACGTCTGCGAGCTGCAGCCC | CTGCAGGGACAGGCGTCAGTGA |
| PKD1 exon13-15 | TGGAGGGAGGGACGCCAATC | GTCAACGTGGGCCTCCAAGT |
| PKD1 exon15-21 | ATCCCTGGGGGTCCTACCATCTCTTA | ACACAGGACAGAACGGCTGAGGCTA |

| | | |
|---|---|---|
| PKD1 exon22-26 | ATGCTTAGTGAGGAGGCTGTGGGGGTCCA | CGCTTAAGGGGAATGGCTTAAACCCG |
| PKD1 exon27-34 | CGGGTCACCGGTTGTGGCA | ATGAGGCTCTTTCCACAGACAACAGAGGTT |
| PKD1 exon35-41 | CAAGAGGCTCAAGAAACTGCCCG | GGGCTGTGGAAGCCGCCTA |
| PKD1 exon42-46 | GAGTAGTTCTCCAGGAGTGCCG | ATTCTGCCTGGCCCTCGGCCTT |

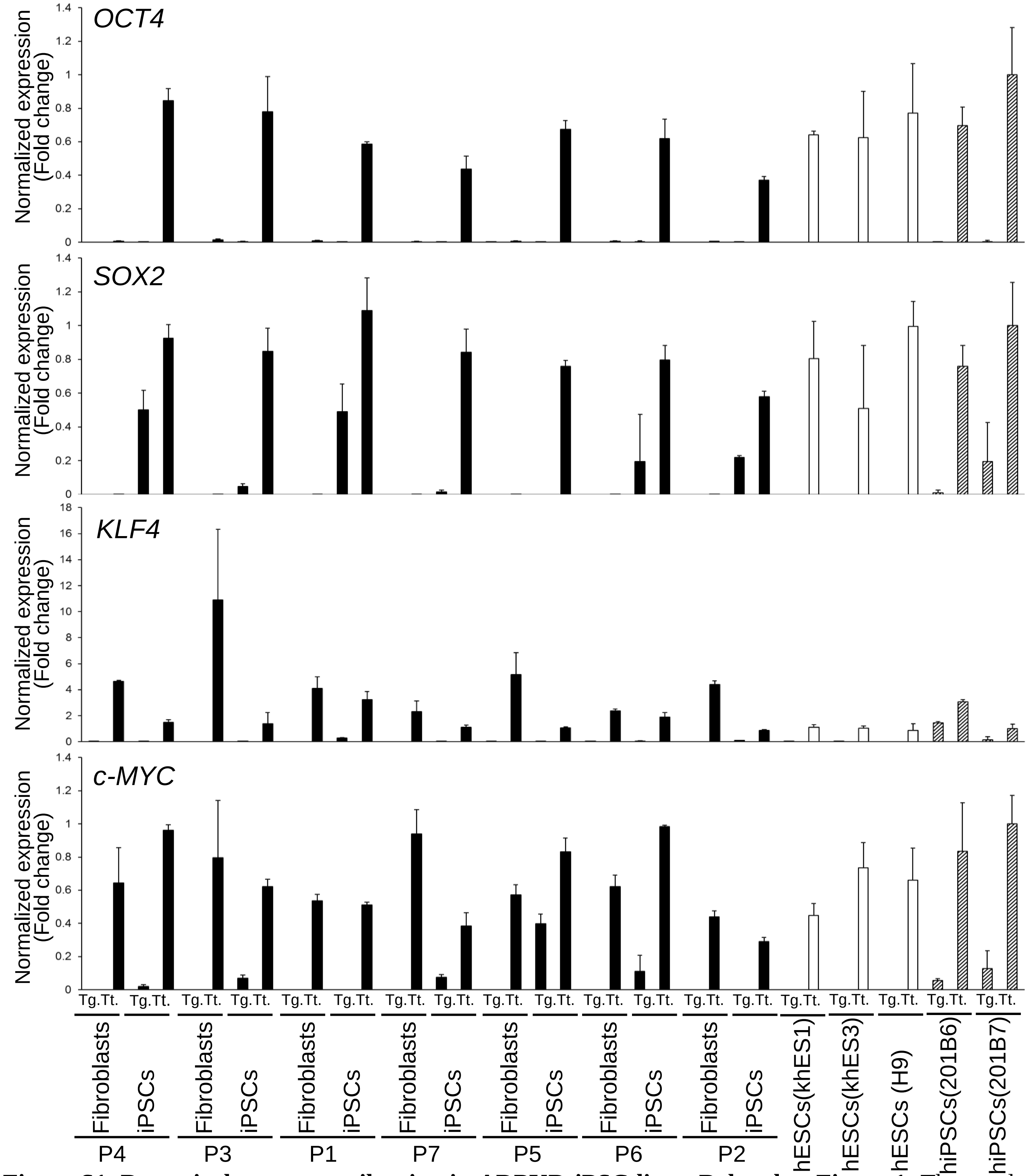


**Figure S1. Retroviral transgene silencing in ADPKD-iPSC lines, Related to Figure 1.** The results of the qRT-PCR analyses for the retroviral transgene (Tg) and total (Tt; the sum of the transgene and endogenous) expression of *OCT4*, *SOX2*, *KLF4* and *c-MYC* in ADPKD-iPSC lines and their parental fibroblasts from all seven patients (P1-7). hESC lines (khES1, khES3 and H9) and hiPSC lines (201B6 and 201B7) were used as controls. Each value was normalized to the samples from 201B7. Note that the expression level of Tg was so low in many samples that the histogram bars are at the baseline. All values are the means ± SD of triplicate samples from three independent experiments.

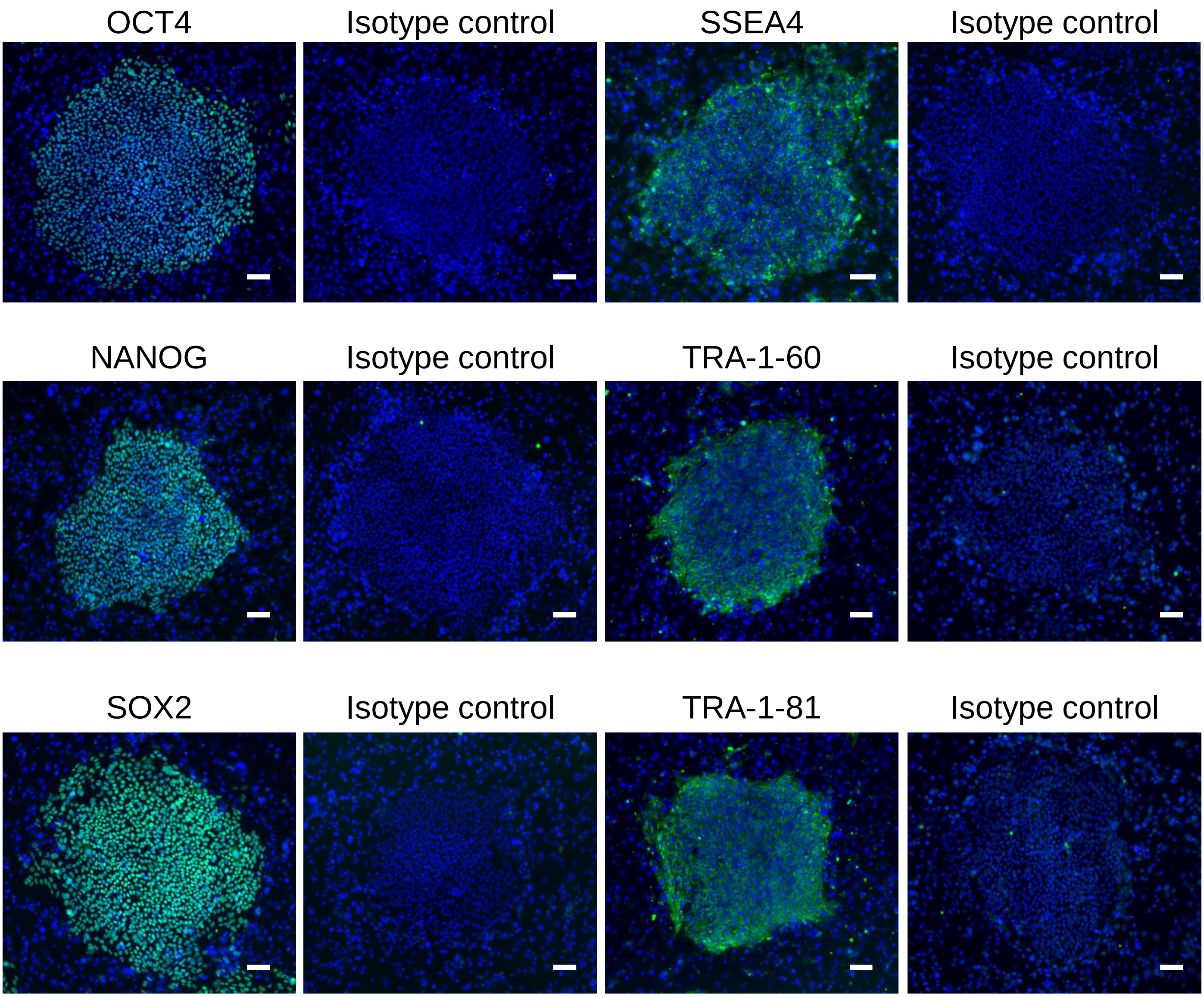


**Figure S2. Expression of pluripotent marker genes, Related to Figure 1.** The expression of pluripotency markers, such as OCT4, NANOG, SOX2, SSEA4, TRA-1-60 and TRA-1-81 in P4-iPSCs. Isotype control antibodies were used at the same concentrations as the tested antibodies. Scale bars, 100 μm.

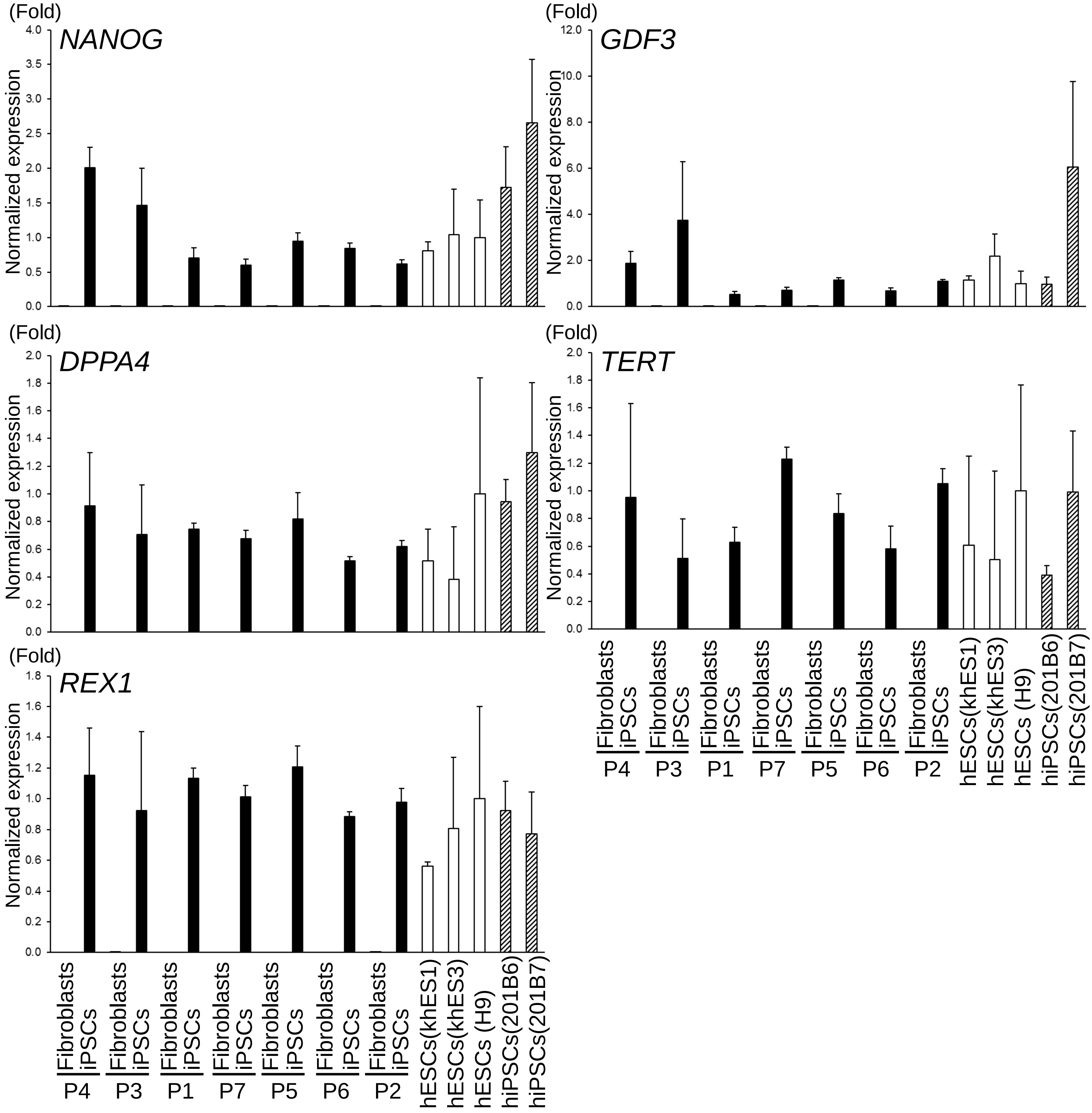


**Figure S3. Gene expression analysis of ADPKD-iPSC lines, Related to Figure 1.** qRT-PCR analyses were used to evaluate the expression of *NANOG*, *DPPA4*, *REX1*, *GDF3* and *TERT* in ADPKD-iPSC lines from all seven patients (P1-7) and their parental fibroblasts. Three hESC lines (H9, khES1 and khES3) and two hiPSC lines (201B6 and 201B7) were used as positive controls for the pluripotency gene expressions. Each value was normalized to the samples from H9. Note that the expression level of the five genes in fibroblast samples was so low that the histogram bars are at the baseline. All values are the means ± SD of triplicate samples from three independent experiments.

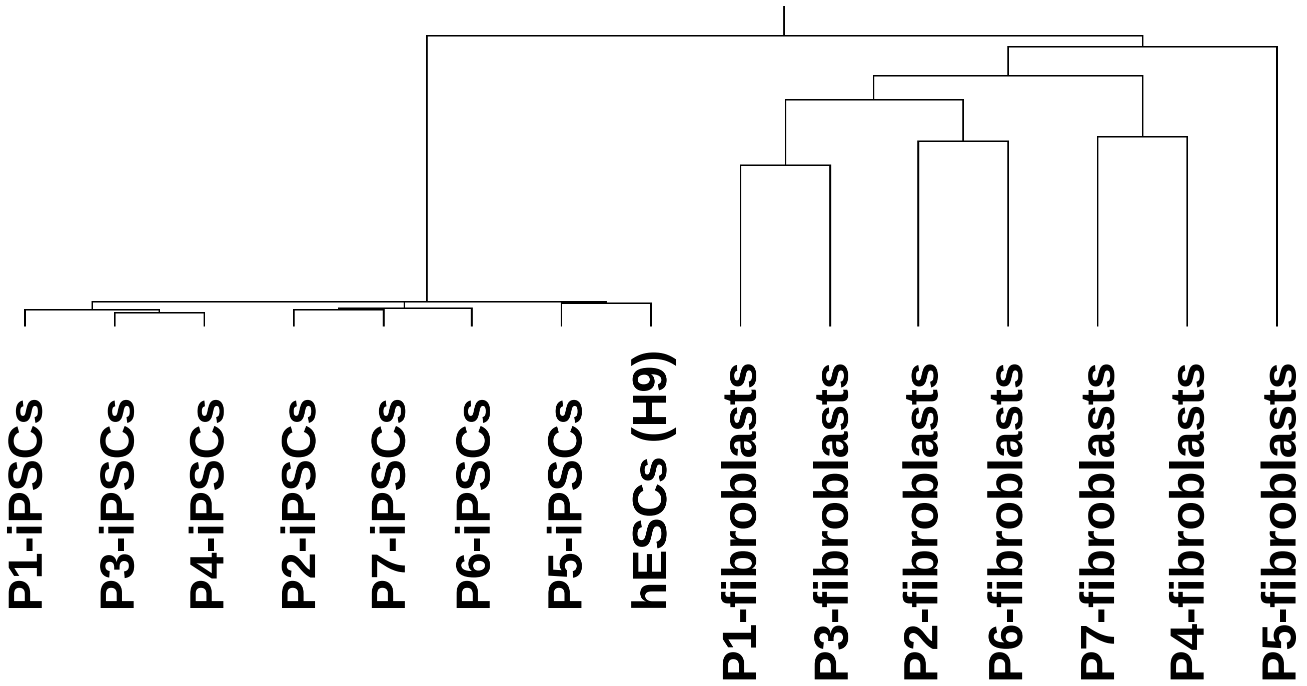


**Figure S4. Global gene expression analyses of ADPKD-iPSC lines, Related to Figure 1.** The microarray analysis and hierarchical clustering showed that the global gene expression profiles of ADPKD-iPSCs were more similar to those of hESCs (H9) than to those of the parental fibroblasts.

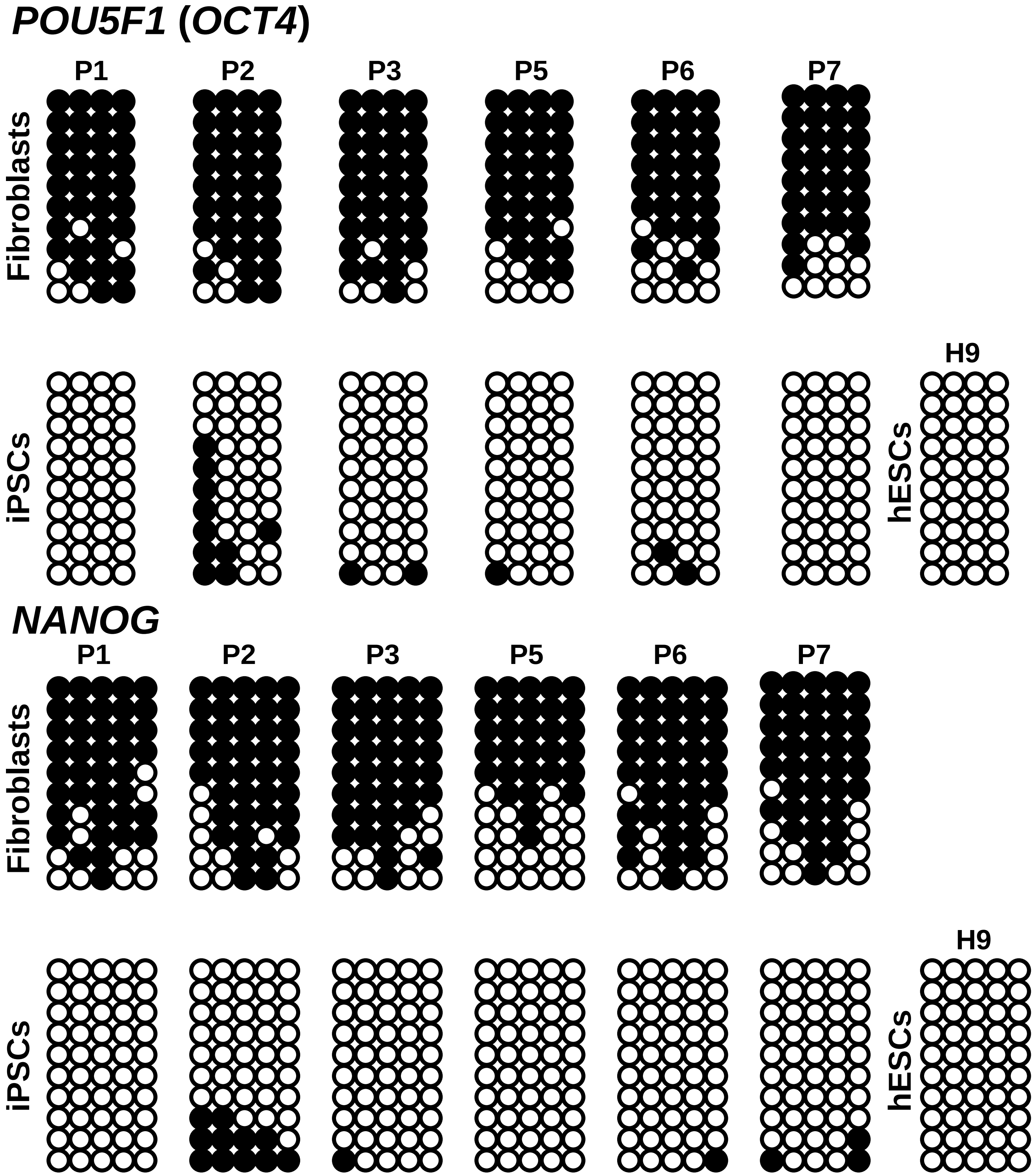


**Figure S5. Methylation analyses of the *OCT4* and *NANOG* promoters in ADPKD-iPSC lines, Related to Figure 1.** Bisulphite sequencing analyses of the *OCT4* and *NANOG* promoters in ADPKD-iPSC lines from six patients (P1-3 and P5-7) and their parental fibroblasts. The hESC line (H9) was used as a control.

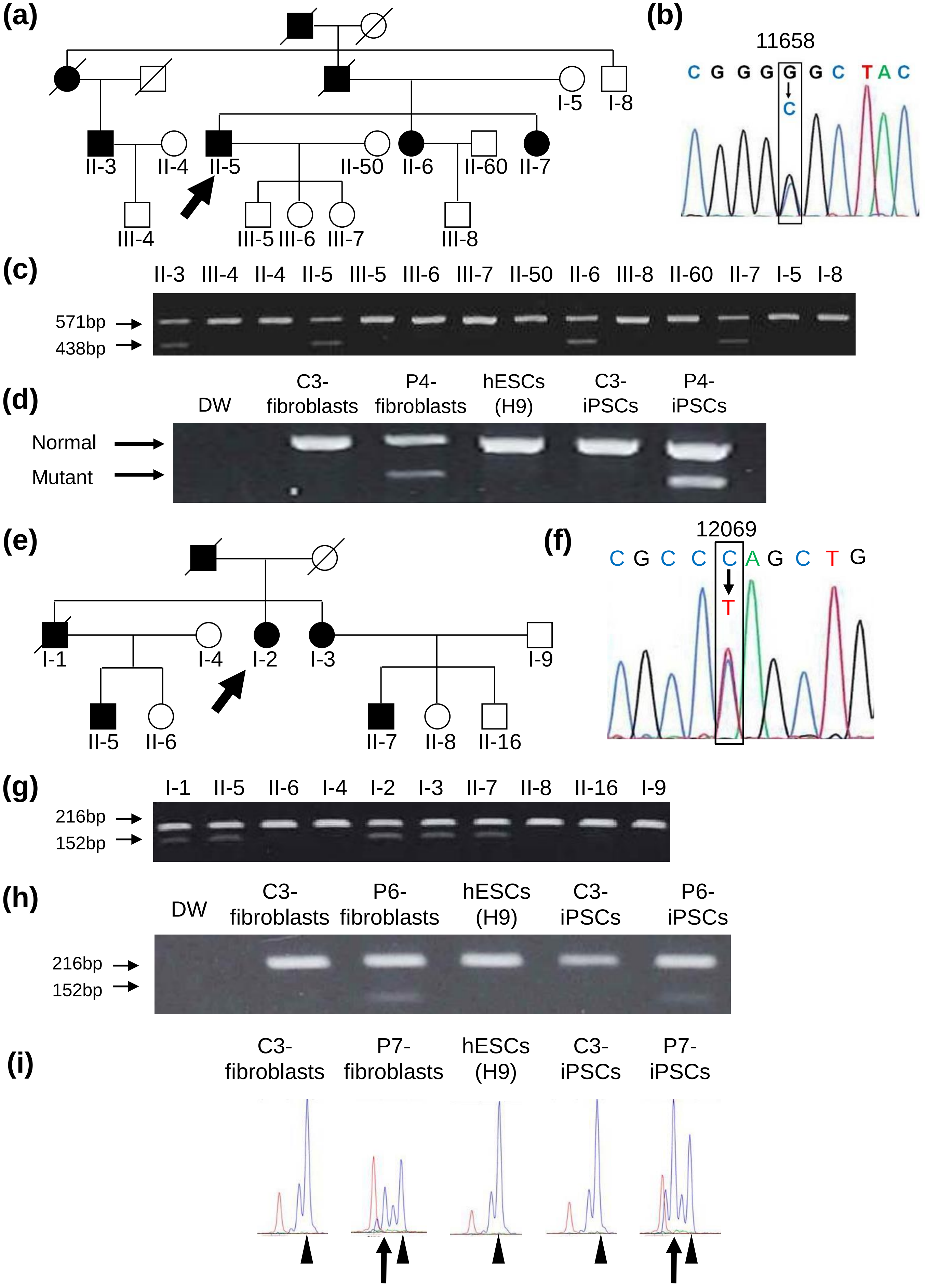
(a)
I-5
I-8
II-3
II-4
II-5
II-50
II-6
II-60
II-7
III-4
III-5
III-6
III-7
III-8
(b)
11658
C G G G G G C T A C
C
(c)
II-3 III-4 II-4 II-5 III-5 III-6 III-7 II-50 II-6 III-8 II-60 II-7 I-5 I-8
571bp
438bp
(d)
DW
C3-fibroblasts
P4-fibroblasts
hESCs (H9)
C3-iPSCs
P4-iPSCs
Normal
Mutant
(e)
I-1
I-4
I-2
I-3
I-9
II-5
II-6
II-7
II-8
II-16
(f)
12069
C G C C C A G C T G
T
(g)
I-1 II-5 II-6 I-4 I-2 I-3 II-7 II-8 II-16 I-9
216bp
152bp
(h)
DW
C3-fibroblasts
P6-fibroblasts
hESCs (H9)
C3-iPSCs
P6-iPSCs
216bp
152bp
(i)
C3-fibroblasts
P7-fibroblasts
hESCs (H9)
C3-iPSCs
P7-iPSCs

**Figure S6. Mutation analyses, Related to Figure 1.** (a) ADPKD patient P4 and pedigree. Solid symbols indicate affected members. The arrow indicates the patient (II-5). (b) Sequencing results showed the presence of 11658G>C mutation in *PKD1* in P4. (c) PCR RFLP verification of the mutation in genomic DNA from fresh peripheral blood from P4 and other members of the pedigree. A RFLP analysis was done using *Bfa*I. (d) PCR RFLP verification of the mutation in P4-iPSCs, P4-fibroblasts, control (C3)-iPSCs, hESCs (H9) and C3-fibroblasts. (e) Patient P6 and pedigree. Solid symbols indicate affected members. The arrow indicates the patient (I-2). (f) The sequencing results showed the presence of 12069C>T mutation in *PKD1* in P6. (g) PCR RFLP verification of the mutation in genomic DNA from fresh peripheral blood from P6 and other members of the pedigree. A RFLP analysis was done using *Hae*II. (h) PCR RFLP verification of the mutation in P6-iPSCs, P6-fibroblasts, C3-iPSCs, H9 and C3-fibroblasts. (i) Detection of a 2-bp deletion (7024del AC) in exon 15 of *PKD1* in patient P7.[1] The expected size of the patient allele was 202 bp (arrows), while the normal allele was 204 bp (arrowheads). Both P7-iPSCs and P7-fibroblasts showed the same genotype of 202/204, while C3-iPSCs, C3-fibroblasts and H9 showed the genotype of 204/204. Red waves indicate 200 bp.

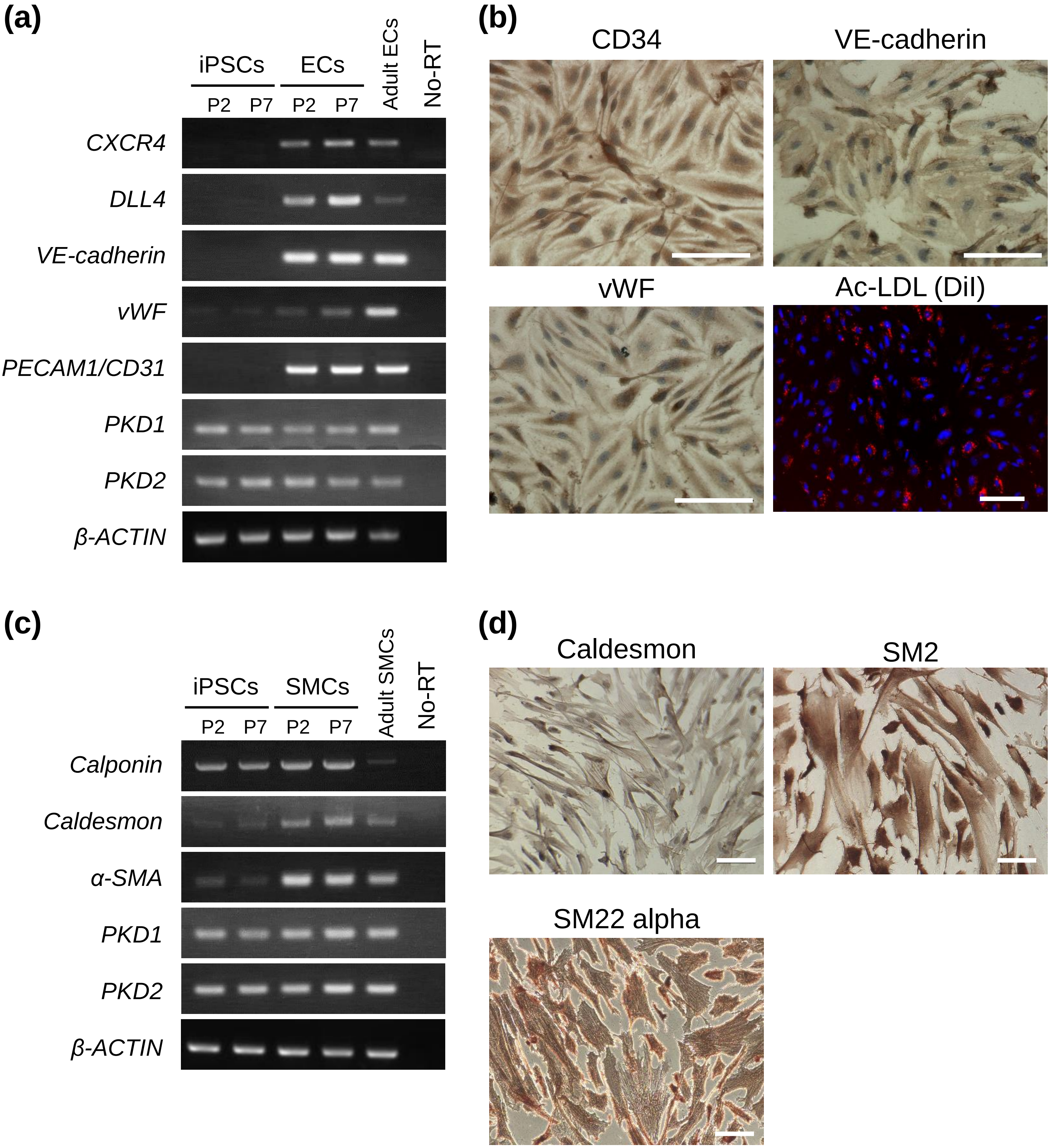
(a)
iPSCs
ECs
Adult ECs
No-RT
P2
P7
P2
P7
CXCR4
DLL4
VE-cadherin
vWF
PECAM1/CD31
PKD1
PKD2
β-ACTIN
(b)
CD34
VE-cadherin
vWF
Ac-LDL (DiI)
(c)
iPSCs
SMCs
Adult SMCs
No-RT
P2
P7
P2
P7
Calponin
Caldesmon
α-SMA
PKD1
PKD2
β-ACTIN
(d)
Caldesmon
SM2
SM22 alpha

**Figure S7. Directed differentiation of ADPKD-iPSCs into vascular endothelia and smooth muscle cells, Related to Figure 2.** (a) The expression of endothelial marker genes (*CXCR4*, *DLL4*, *VE-cadherin*, *vWF* and *PECAM1/CD31*) and *PKD1* and *PKD2* in vascular endothelia derived from two ADPKD-iPSC lines (P2- and P7-iPSCs). (b) Immunostaining of vascular endothelia on culture day 12 differentiated from P4-iPSCs for endothelial markers, CD34, VE-cadherin and von Willebrand factor (vWF) , and the uptake of DiI labeled acetylated low density lipoprotein (DiI-Ac-LDL). (c) The expression of smooth muscle cell marker genes (*Calponin*, *Caldesmon* and *a-SMA*) and *PKD1* and *PKD2* in vascular smooth muscle cells derived from two ADPKD-iPSC lines (P2- and P7-iPSCs). (d) Immunostaining of vascular smooth muscle cells on culture day 25 differentiated from P4-iPSCs for smooth muscle cell markers, caldesmon, smooth muscle myosin heavy chain (SM2) and SM22 alpha. Scale bars, 100 μm. ECs, endothelial cells; SMCs, smooth muscle cells.

**(a)**

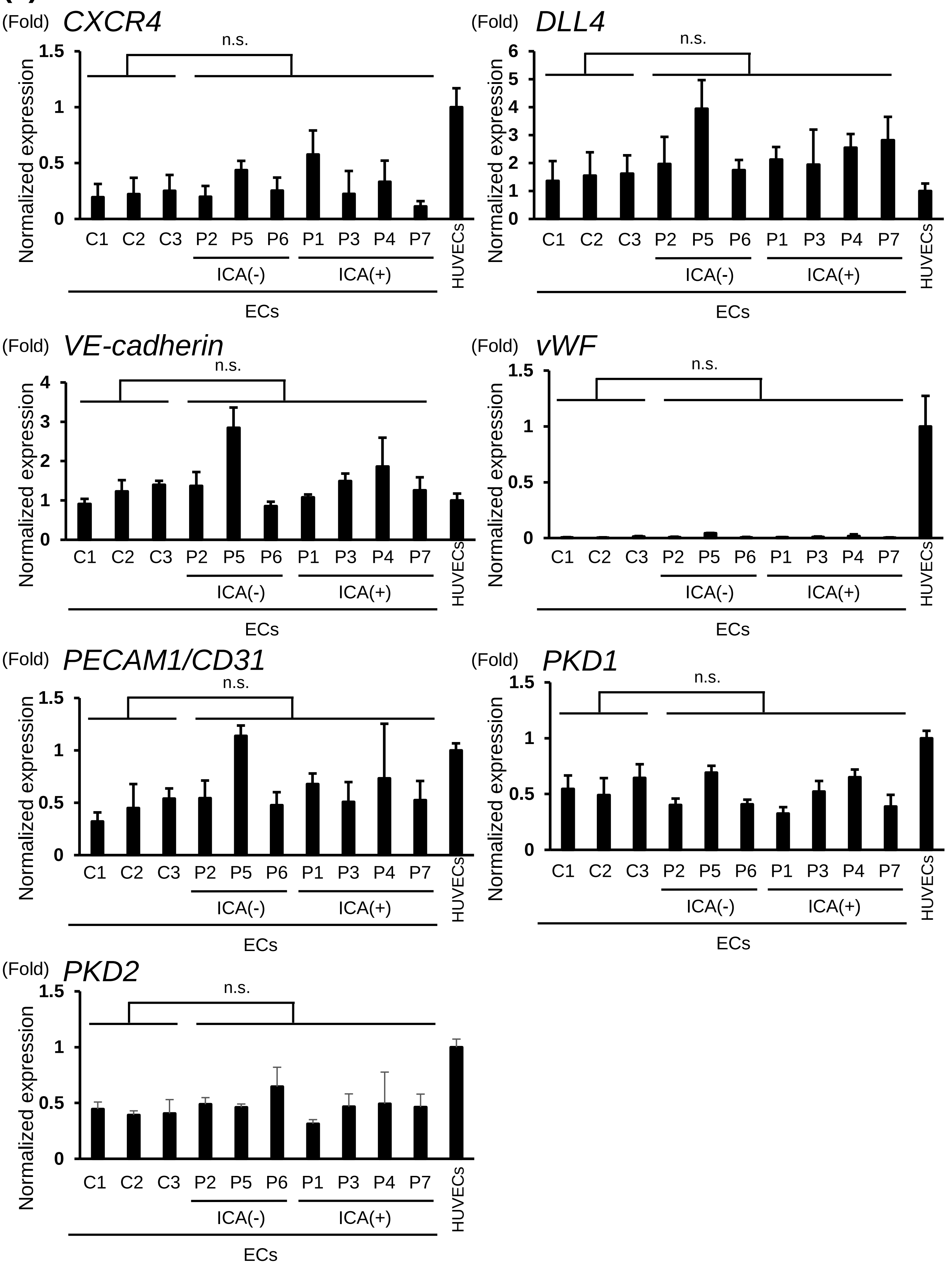

(Fold) CXCR4
n.s.
1.5
1
0.5
0
Normalized expression
C1 C2 C3 P2 P5 P6 P1 P3 P4 P7
ICA(-) ICA(+)
ECs
HUVECs
(Fold) DLL4
n.s.
6 5 4 3 2 1 0
(Fold) VE-cadherin
n.s.
4 3 2 1 0
(Fold) vWF
n.s.
1.5 1 0.5 0
(Fold) PECAM1/CD31
n.s.
1.5 1 0.5 0
(Fold) PKD1
n.s.
1.5 1 0.5 0
(Fold) PKD2
n.s.
1.5 1 0.5 0

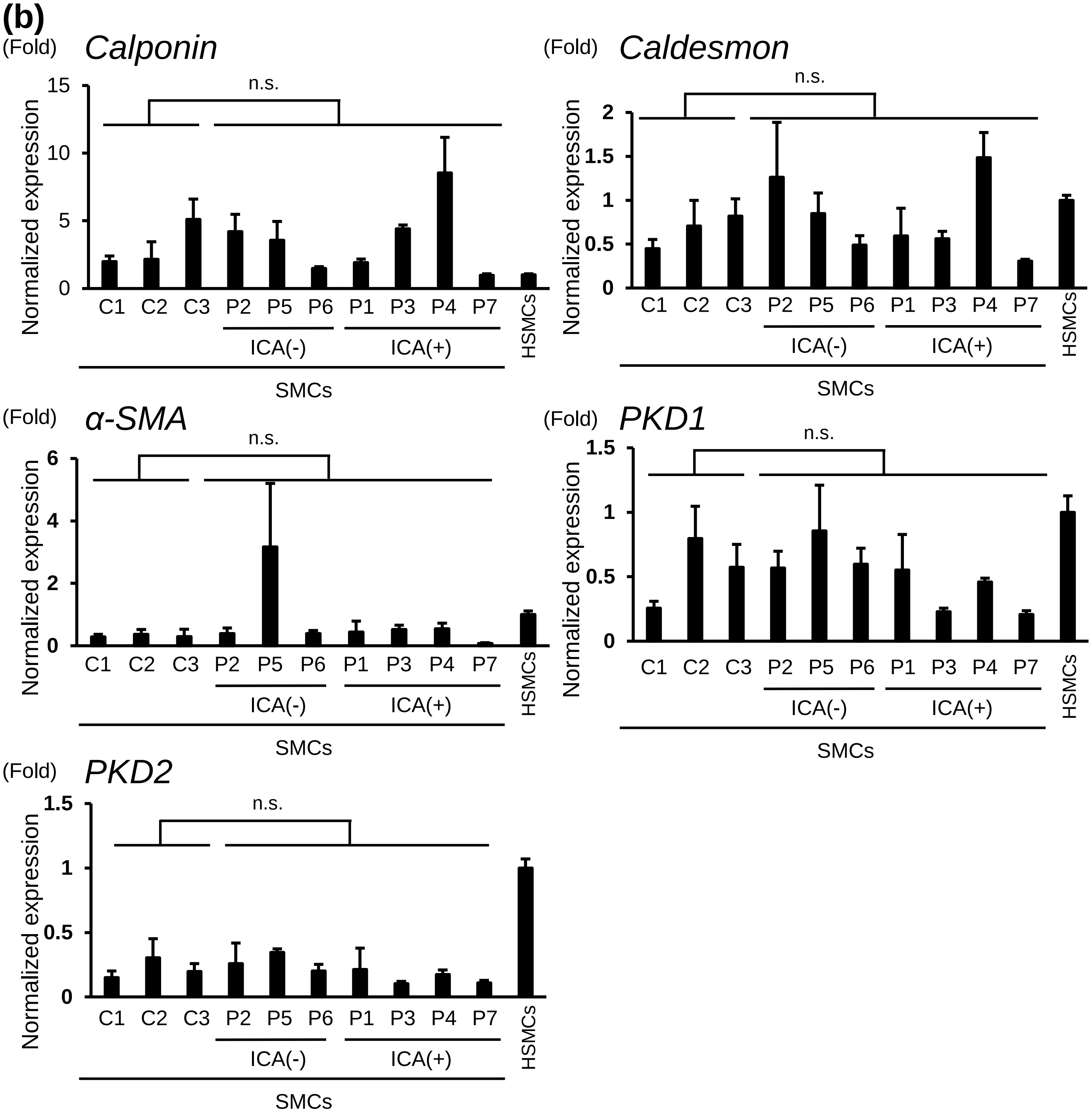


**Figure S8. Comparison of marker gene expression between vascular cells derived from control- and ADPKD-iPSCs, Related to Figure 2.** (a, b) qRT-PCR analyses were used to compare the expression of marker genes for vascular endothelia (a) and smooth muscle cells (b) and of *PKD1* and *PKD2* between endothelia (a) and smooth muscle cells (b) differentiated from control- and ADPKD-iPSCs. Each value was normalized to the average of those of HUVEC samples in (a) and HSMC samples in (b). All values are presented as the means ± SD of triplicate samples from three independent experiments. ECs, endothelial cells; SMCs, smooth muscle cells; ICA, intracranial aneurysms.

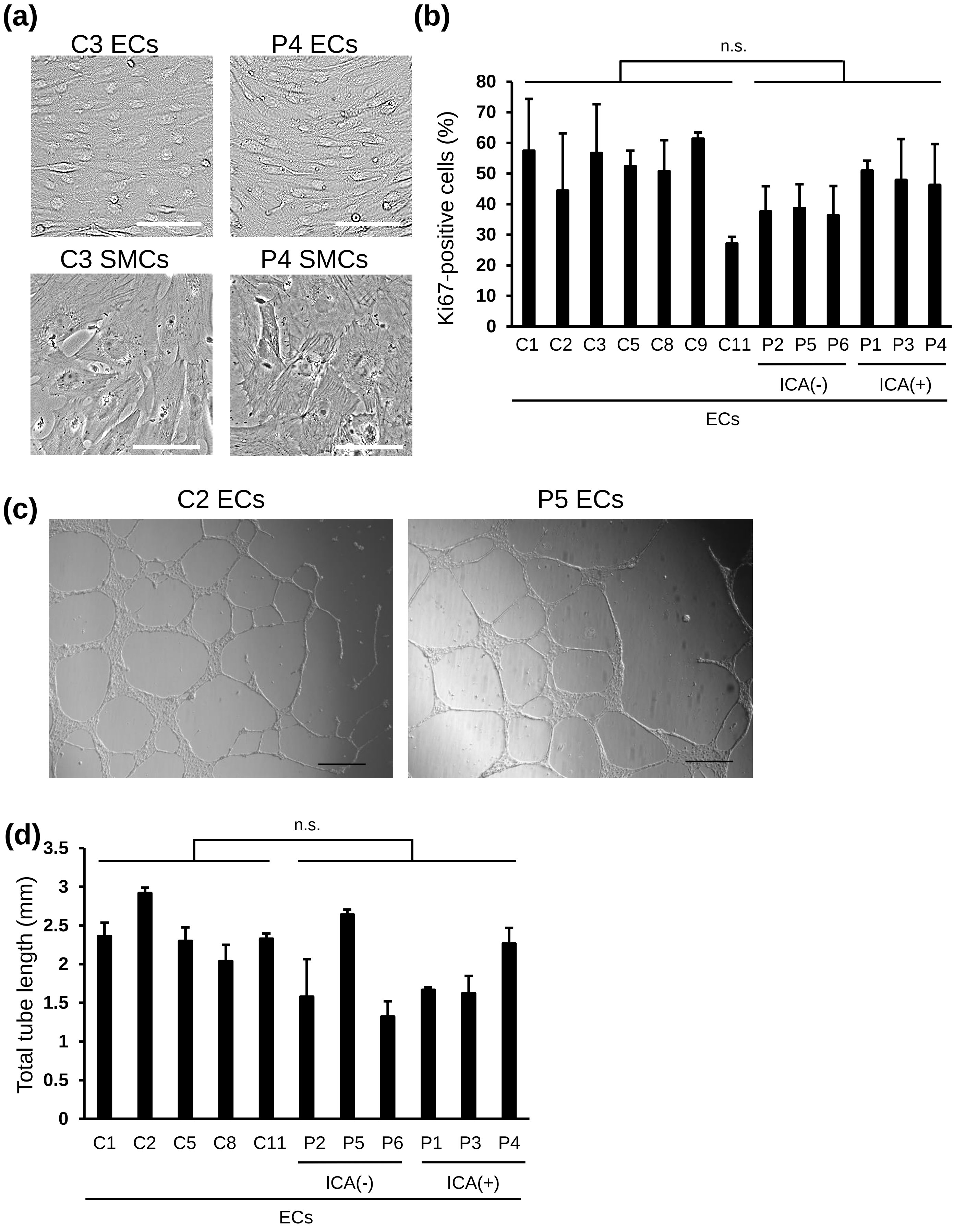
(a)
C3 ECs
P4 ECs
C3 SMCs
P4 SMCs
(b)
n.s.
Ki67-positive cells (%)
0
10
20
30
40
50
60
70
80
C1 C2 C3 C5 C8 C9 C11 P2 P5 P6 P1 P3 P4
ICA(-)
ICA(+)
ECs
(c)
C2 ECs
P5 ECs
(d)
n.s.
Total tube length (mm)
0
0.5
1
1.5
2
2.5
3
3.5
C1 C2 C5 C8 C11 P2 P5 P6 P1 P3 P4
ICA(-)
ICA(+)
ECs

**Figure S9. Comparison of proliferation and angiogenic potentials between vascular endothelia derived from control- and ADPKD-iPSCs, Related to Figure 2.** (a) No obvious morphological differences were found between vascular cells differentiated from control- and ADPKD-iPSCs. Typical images of endothelial cells (ECs) and smooth muscle cells (SMCs) differentiated from control (C3)- and ADPKD (P4)-iPSCs. (b) Proliferation potential of hiPSC-derived endothelia was assessed by quantitative analyses of Ki-67 immunostaining images. (c, d) Tube formation assay was performed to evaluate the angiogenic activity of hiPSC-derived endothelia. Typical images of endothelia differentiated from control (C2)- and ADPKD (P5)-iPSCs. (d) Total tube length was calculated as the average of three fields. All values are the means ± SD of triplicate samples from three independent experiments. Scale bars, 100 μm in (a) and 500 μm in (c). ICA, intracranial aneurysms; n.s., not significant.

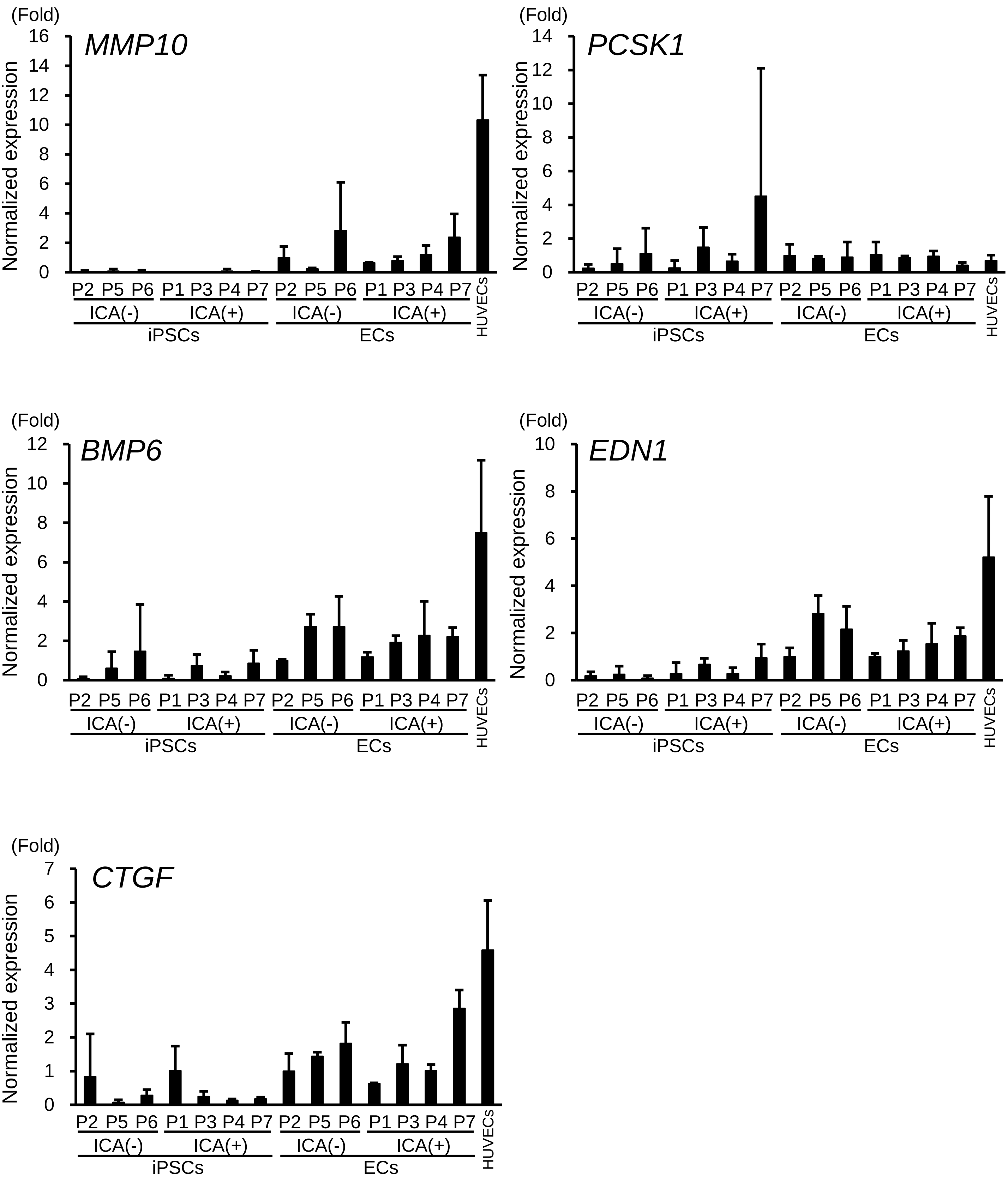

(Fold)
MMP10
Normalized expression
P2 P5 P6 P1 P3 P4 P7 P2 P5 P6 P1 P3 P4 P7
ICA(-) ICA(+) ICA(-) ICA(+)
iPSCs
ECs
HUVECs
(Fold)
PCSK1
Normalized expression
P2 P5 P6 P1 P3 P4 P7 P2 P5 P6 P1 P3 P4 P7
ICA(-) ICA(+) ICA(-) ICA(+)
iPSCs
ECs
HUVECs
(Fold)
BMP6
Normalized expression
P2 P5 P6 P1 P3 P4 P7 P2 P5 P6 P1 P3 P4 P7
ICA(-) ICA(+) ICA(-) ICA(+)
iPSCs
ECs
HUVECs
(Fold)
EDN1
Normalized expression
P2 P5 P6 P1 P3 P4 P7 P2 P5 P6 P1 P3 P4 P7
ICA(-) ICA(+) ICA(-) ICA(+)
iPSCs
ECs
HUVECs
(Fold)
CTGF
Normalized expression
P2 P5 P6 P1 P3 P4 P7 P2 P5 P6 P1 P3 P4 P7
ICA(-) ICA(+) ICA(-) ICA(+)
iPSCs
ECs
HUVECs

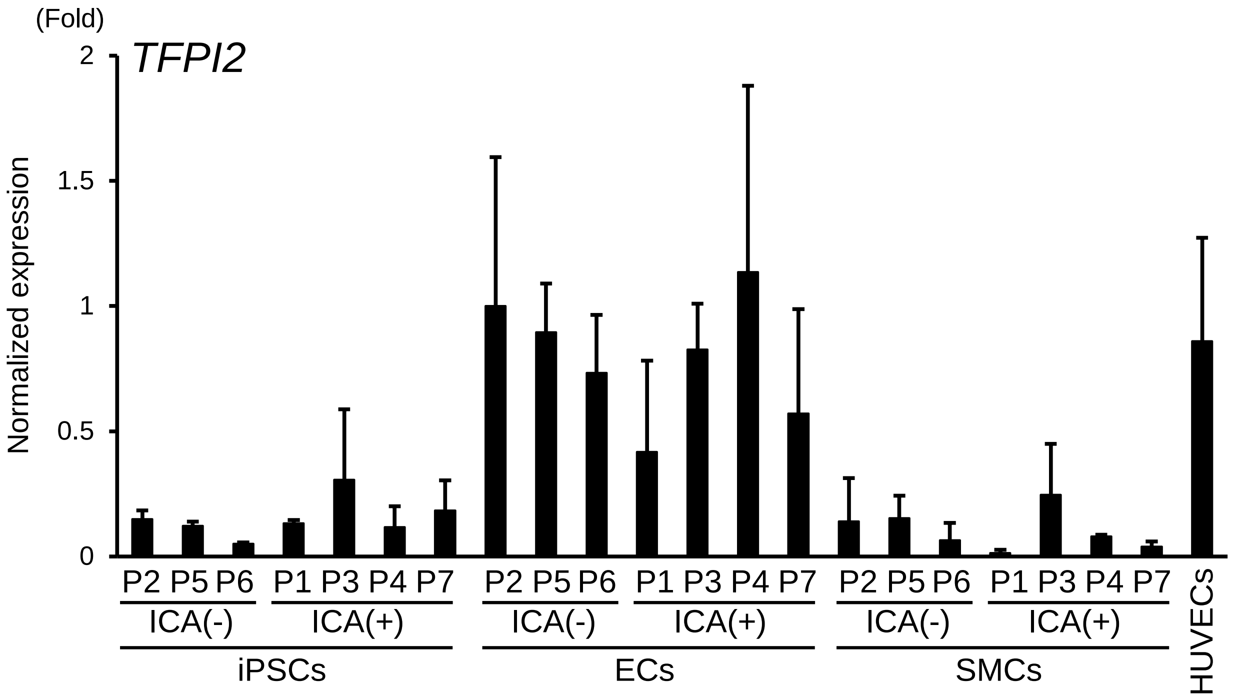


**Figure S10. Gene expression analyses of iPSC-derived endothelia and smooth muscle cells from ADPKD patients with intracranial aneurysms (ICAs) and those without ICAs, Related to Figure 5.** qRT-PCR validation of the microarray data for the expression of *MMP10*, *PCSK1*, *BMP6*, *EDN1* and *CTGF* in ADPKD-iPSCs and iPSC-derived endothelial cells (ECs), and that of *TFPI2* in ADPKD-iPSCs and iPSC-derived ECs and smooth muscle cells (SMCs) from the ADPKD patients with ICAs (P1, P3, P4 and P7) compared with the patients without ICAs (P2, P5 and P6). Each value was normalized to the average of iPSC-derived EC samples from P2. All values are the means ± SD of triplicate samples from three independent experiments. Note that the iPSC-derived ECs induced by our differentiation protocol have features of arterial ECs and therefore the expression profiles of some genes were different between iPSC-derived arterial ECs and human umbilical vein ECs (HUVECs).[2,3] ICA, intracranial aneurysms.

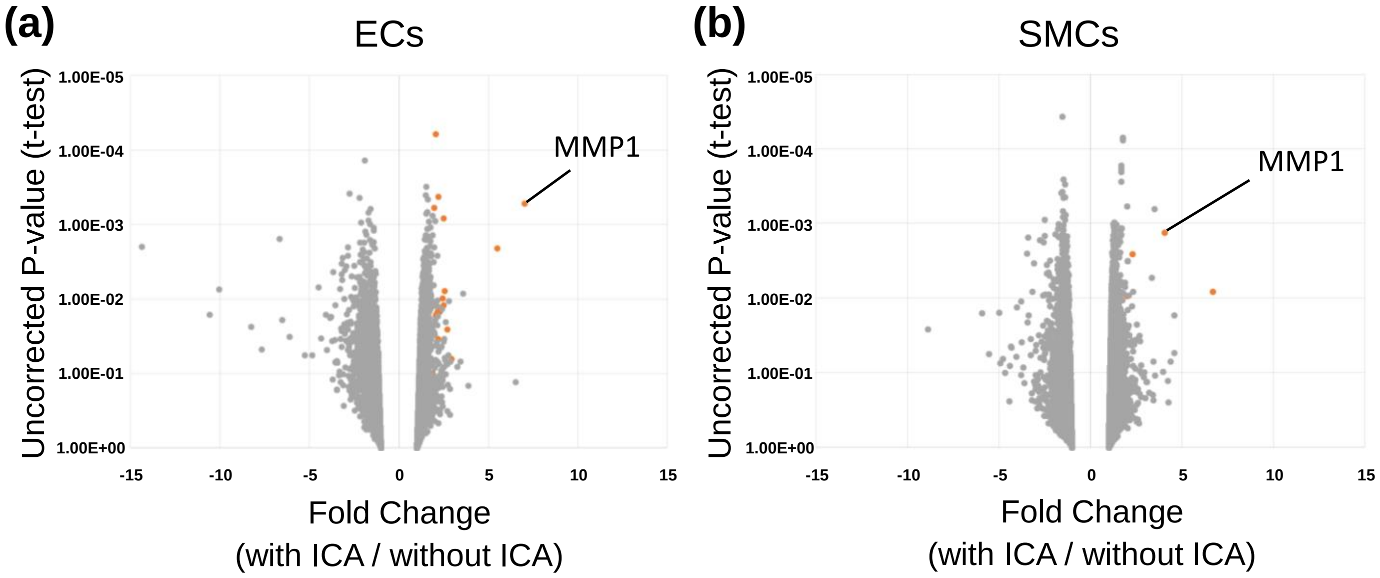


**Figure S11. Comparison of gene expression profiles of iPSC-derived vascular cells from ADPKD patients with intracranial aneurysms (ICAs) and those from ADPKD patients without ICAs, Related to Figure 5.** (a, b) Volcano plots were drawn with fold changes (with ICAs/without ICAs) and uncorrected *P*-values (t-test) of the microarray data for vascular endothelia (a) and smooth muscle cells (b). Orange plots were the candidate genes that are listed in **Tables S6** and **S7**. ECs, endothelial cells; SMCs, smooth muscle cells.

## Supplementary Materials and Methods

### Plasmid Construction and Lentivirus/Retrovirus Production.

The pMXs-based retroviral vectors for human *OCT4*, *SOX2*, *KLF4* and *c-MYC* have been described previously.[4] A 20 bp random sequence, which was designated the $N_{20}$ barcode, was introduced into the *Not*I/*Sal*I site of the *OCT4* expression vector. This unique barcode sequence was used in each experiment to avoid inter-experimental contamination. Supernatant containing the pMX retroviruses was generated by transfecting PLAT-E packaging cells with the pMXs vectors using the Fugene 6 transfection reagent (Roche).

The mouse *Slc7a1* ORF was transferred to pLenti6/UbC/V5-DEST (Thermo Fisher Scientific) by the Gateway cloning system (Thermo Fisher Scientific). Supernatant containing the mouse Slc7a1 lentiviruses was produced by transfecting 293FT cells (Thermo Fisher Scientific) with pLenti6/UbC-Slc7a1 and Virapower packaging mix using Lipofectamine 2000 (Thermo Fisher Scientific).

### Generation of Patient-specific iPSCs

Fibroblasts derived from ADPKD patients were maintained and expanded in Dulbecco's

Modified Eagle Medium (DMEM, Nacalai Tesque) containing 10% fetal bovine serum (FBS, Japan Bioserum). The induction of iPSCs was performed as described previously.[4] In brief, the patients' fibroblasts were seeded in six-well plates at $1.0 \times 10^5$ cells/well. The next day, the cells were infected with Slc7a1 lentiviruses with 4 μg/ml polybrene (Nacalai Tesque). Then, the patients' fibroblasts expressing the mouse *Slc7a1* gene were seeded in six-well plates at $1.0 \times 10^5$ cells/well one day before transduction. Equal amounts of three or four retrovirus-containing supernatants were mixed and supplemented with 4 μg/ml polybrene. Six days after transduction, the fibroblasts were replated onto mitomycin C–treated SNL feeder cells.[5] Thirty days after transduction, iPSC colonies were selected for expansion.

**Cell Culture**

iPSCs were maintained in Primate ES medium (ReproCELL) supplemented with 500 U/ml penicillin/streptomycin (Thermo Fisher Scientific) and 4 ng/ml recombinant human basic fibroblast growth factor (bFGF, WAKO). For routine passaging, human iPSCs were washed once with phosphate buffered saline (PBS, Nacalai Tesque) and then incubated with CTK dissociation solution containing 0.1% collagenase IV, 0.25% trypsin, 20% knockout serum replacement (KSR, Thermo Fisher Scientific) and 1 mM

$CaCl_2$ in PBS. The split ratio was routinely between 1:3 and 1:6.

**RT-PCR and Real-time Quantitative RT-PCR (qRT-PCR)**

Total RNA was isolated using the RNeasy kit (Qiagen) according to the manufacturer's recommendations, followed by cDNA synthesis using standard protocols. Briefly, 1 μg of total RNA was treated with DNase I (Qiagen) for 15 min, and the cDNA was synthesized using ReverTra Ace (TOYOBO). The cDNA samples were subjected to PCR amplification using a thermal cycler (Veriti 96-well Thermal Cycler, Thermo Fisher Scientific). PCR was performed using the Ex-Taq PCR kit (Takara) according to the manufacturer's instructions. The PCR cycles were as follows: for *β-ACTIN*, initial denaturation was performed at 94°C for 2.5 min, followed by 25 cycles of 94°C for 30 s, 60°C for 1 min, 72°C for 30 s and a final extension at 72°C for 10 min. For the other genes, the cycles consisted of initial denaturation at 94°C for 2.5 min, followed by 30-40 cycles of 94°C for 30 s, 58-62°C for 30 s, 72°C for 30 s and a final extension at 72°C for 7 min. qPCR was performed using the Step One Plus Real-Time PCR System (Thermo Fisher Scientific) and SYBR Green PCR Master Mix (Takara). Denaturation was performed at 95°C for 10 min, followed by 45 cycles at 95°C for 5 s and at 60°C for 30 s. As recommended by the manufacturer, the threshold cycle method was used to

analyze the data for the gene expression levels and was normalized to those of the housekeeping gene, *β-ACTIN*. The PCR reactions were performed in triplicate for each sample. The primer sequences are listed in **Table S10**.

**Short Tandem Repeat (STR) Analysis and Karyotyping**

The STR analyses were performed at BEX CO. LTD., Japan, and the chromosomal G-band analyses were performed at the Nihon Gene Research Laboratories, Japan.

**Bisulfite Sequencing**

Sodium bisulfite conversion of genomic DNA (1 μg) was performed using the EZ DNA methylation kit (ZYMO Research), according to the manufacturer’s instructions. The promoter regions of the human *OCT4* and *NANOG* genes were amplified by PCR as described previously.[4] The PCR products were subcloned into the pCR4-TOPO TA vector (Thermo Fisher Scientific) and were sequenced.

**Embryoid Body (EB) Formation**

For EB formation, a 10 cm plate containing human iPSCs was rinsed with PBS and treated with 1 mg/ml type IV collagenase (Thermo Fisher Scientific) in DMEM for 10

min at 37°C. The collagenase was rinsed away with PBS and replaced with undifferentiation medium. The cells were then scraped off with a cell scraper (IWAKI), dissociated by pipetting, and distributed into a low attachment 6-well plate (Corning) containing knockout-DMEM (Thermo Fisher Scientific) supplemented with 20% KSR, 0.1 mM non-essential amino acids (Thermo Fisher Scientific), 2 mM glutamine (Thermo Fisher Scientific), 500 U/ml penicillin/streptomycin and 0.55 mM 2-mercaptoethanol (Thermo Fisher Scientific). After 8 days as a floating culture, the EBs were transferred to gelatin-coated plates and cultured in the same medium for another 8 days.

**Teratoma Formation**

The iPSCs were harvested using CTK solution, collected into tubes and centrifuged, and the pellets were resuspended in DMEM/F12 (Thermo Fisher Scientific). One quarter of the cells from a confluent 100 mm dish was injected into the testes of a NOD-SCID mouse (CREA). Nine to twelve weeks after injection, the tumors were dissected and fixed with PBS containing 4% paraformaldehyde (PFA). Paraffin-embedded tissues were sliced and stained with hematoxylin and eosin.

**Mutational Analysis**

Mutational analyses of the three families with ADPKD (P4, P6 and P7) were performed as described previously, with some modifications.[1] In brief, the pedigree structures were constructed based on interviews, and additional medical histories were obtained from patient records. Informed consent, in accordance with Kyoto University Hospital guidelines, was obtained from each patient and/or their family. Multipoint linkage analyses and haplotype constructions were performed to confirm the prevalent type of ADPKD as *PKD1* in all the three pedigrees using four microsatellite markers, D16S404, D16S3075 (*PKD1*), D16S103 and D16S3046, spanning 27 cM on chromosome 16p. To exclude linkage with the *PKD2* locus, we used six microsatellite markers, D4S392, D4S2964, D4S1534 (*PKD2*), D4S414, D4S1562 and D4S406, spanning 38.7 cM on chromosome 4q. Linkage studies were performed using GENEHUNTER software.[6] The *PKD1* genomic DNA was isolated from either fresh or frozen peripheral blood. The DNA was extracted using two kinds of commercially available extraction kits, the Wizard Genomic DNA Purification Kit (Promega) and the QIAamp DNA Blood Mini Kit (Qiagen). The primer pairs used to amplify the *PKD1* (GenBank accession number L39891) genomic fragments are shown in **Table S10**. PCR amplification was performed in a PTC-100 programmable thermal controller (MJ Research) or in a Gene

Amp PCR System 9700 (Thermo Fisher Scientific). For the mutational analysis of the duplicated part of *PKD1*, long-range PCR (LR-PCR) followed by nested primer PCR was performed. LR-PCR was carried out using a TaKaRa LA PCRTM Kit Ver. 2.1 (Takara) with a hot start protocol, *PKD1*-specific primers (**Table S10**), and protocols that were reported previously.[7-10] For nested PCR after LR-PCR, diluted (1/105) product was used to avoid any genomic influence. The GC-rich region was amplified using the GC buffer provided in the TaKaRa LA PCRTM Kit Ver. 2.1. The quality of the PCR-amplified products was assessed by agarose-gel electrophoresis. The bands were excised and purified using a Prep-A Gene DNA Purification Kit (Bio-Rad) or a QIAquick Gel Extraction kit (Qiagen). An ABI PRISM dRhodamine terminator cycle sequencing ready reaction kit (Thermo Fisher Scientific) was used to sequence the purified PCR products, and automated sequencing was performed using an ABI PRISM 310 genetic analyzer (Thermo Fisher Scientific).

To elucidate the 2 bp deletion mutation of the family of patient P7, genotyping was performed using the GeneScan™ device (Thermo Fisher Scientific), which can detect an allele two bases shorter by semiautomatic electrophoresis of the PCR-amplified products using fluorescently-labeled primers. For patient P7, the 2 bp deletion in exon 15 of *PKD1* was amplified by LR-PCR, followed by nested-PCR. The

primer sets used were reported previously.[5] Briefly, in the LR-PCR, a 3378 bp product was amplified with the F26 and R2LR primers (**Table S10**) and then the product was diluted to $10^{-5}$. The diluted mixture was amplified with the nested primer set of PKDeX15.14F and PKDeX15.14R (**Table S10**). The expected size of the patient allele (7024del AC) was 202, while that of normal size was 204. The difference in size between the mutated allele and the wild allele was identified by the GeneScan™ system using the fluorescently-labeled reverse primer (PKDex15.14R).

The mutational analyses of the remaining four ADPKD patients (P1, P2, P3 and P5) were performed by whole-exome sequencing and amplicon-sequencing.

**Whole-exome Sequencing**

Exon capture and Illumina's library preparation were performed using the SeqCap EZ v3 (Roche) and a TruSeq DNA Sample Preparation Kit (Illumina, San Diego, CA) as reported previously.[11] Paired-end sequencing for 2 X 101 cycles was performed using a TruSeq SBS Kit v3 and a HiSeq2500 instrument (Illumina). Processing of the raw data, mapping and SNV/indel calling were performed as previously reported.[11]

**Amplicon Sequencing**

PCR was performed using KAPA HiFi Hot Start Ready (KAPA Biosystems) for exon 1 of PKD1 or Prime STAR GXL DNA polymerase (TaKaRa, Japan) for the other regions. The PCR conditions when using KAPA HiFi Hot Start was 1 cycle of 95˚C for 5 min, followed by 25 cycles of 98˚C for 20 sec, 65˚C for 15 sec and 72˚C for 3 min. The PCR using Prime STAR GXL was 30 cycles of 98˚C for 10 sec and 68˚C for 30 min. The primer pairs used to amplify the *PKD1* genomic fragments are shown in **Table S10**. PCR products were then purified using a QIAquick PCR Purification Kit (QIAGEN) and Illumina's libraries were prepared using a TruSeq PCR-free Kit (Illumina). Paired-end sequencing for 2 X 151 cycles was performed using a MiSeq device (Illumina). Base calling was done by MiSeq Reporter, and quality filtering was performed by the Qcleaner software program (Amelieff Corporation) with option –qp 20,80 –n 5 --trim 20 –length 32. The sequenced reads were mapped to the region encoding the *PKD1* gene by BWA-MEM 0.7.10. The Genome Analysis Toolkit (GATK) v1.6 software program performed local re-alignment around indels, recalibration and SNVs/indel calling according to the Broad Institute's best-practice v3 guidelines [https://www.broadinstitute.org/gatk/guide/best-practices]. The Autosomal Dominant Polycystic Kidney Disease Mutation Database: PKDB (http://pkdb.mayo.edu/) was used to annotate SNVs/indels.

**Immunocytochemistry and Alkaline Phosphatase Analysis**

Alkaline phosphatase (AP) staining was performed using the Alkaline Phosphatase Detection Kit (Millipore). For immunocytochemistry, cells were fixed with 4% PFA/PBS at 4°C for 20 min after a brief wash with PBS. Fixed cells were washed with PBS and then blocked with PBST (PBS/0.1% Triton X-100)/5% normal donkey serum (Jackson ImmunoResearch Laboratories, JIRL) for 1 h at room temperature. The primary antibodies were diluted in blocking solution and incubated overnight at 4°C. The secondary antibodies were incubated for 1 h at room temperature. The following primary antibodies and dilutions were used: βIII-tubulin (1:1000, MRB-435P, Covance Research Products), α-smooth muscle actin (1:500, A2547, Sigma), Vimentin (1:100, SC-6260, Santa Cruz Biotechnology), SOX17 (1:500, AF1924, R&D systems), OCT3/4 (1:200, AF1759, R&D systems), NANOG (1:200, 4903, Cell Signaling Technology), SOX2 (1:200, 3579, Cell Signaling Technology), SSEA1 (1:200, MAB2155, R&D systems), SSEA4 (1:200, MAB1435, R&D systems), TRA-1-60 (1:200, MAB4360, Millipore), TRA-1-81 (1:200, MAB4381, Millipore), CD31/PECAM1 (1:200, 14-0319-80, eBioscience), eNOS (1:200, 610296, BD Biosciences), Calponin (1:200, M3556, DAKO), CD34 (1:500, GTX28158, GeneTex), VE-cadherin (1:200, 560411,

BD Biosciences), Von Willebrand Factor (1:25, M061601, Dako), Caldesmon (1:100, sc-7574, Santa Cruz Biotechnology), Smooth Muscle Myosin Heavy Chain (1:180, 7601, YAMASA), SM22 alpha (1:200, ab14106, abcam), acetyl-α-tubulin (1:500, 5335, Cell Signaling Technology) and Ki67 (1:1000, NCL-Ki67p, Leica Biosystems). Purified mouse IgG, rabbit IgG and goat IgG whole molecules (Jackson ImmunoResearch) were used as negative controls. For immunocytochemical detection of αSMA, calponin, CD34, VE-cadherin, vWF, caldesmon, SM2 and SM22 alpha, cells were treated using a Dako Cytomation Envision+System–HRP(DAB) kit after incubation with primary antibodies, following the manufacturer’s protocol (DakoCytomation). Nuclei were visualized by hematoxylin staining (Muto Pure Chemicals). For immunocytochemical detection of other antibodies, the secondary antibodies included Alexa488-conjugated donkey anti-mouse IgG, Alexa594-conjugated donkey anti-mouse IgG, Alexa488-conjugated donkey anti-rabbit IgG, Alexa488-conjugated donkey anti-goat IgG, Alexa594-conjugated donkey anti-goat IgG and Alexa594-conjugated donkey anti-rat IgG (1:1000, Thermo Fisher Scientific). Nuclei were stained with Hoechst 33342 (1:1000, Thermo Fisher Scientific). The rate of Ki67 positive cells were examined using In Cell Analyzer 6000 (GE Healthcare).

**DiI Labeled Acetylated Low Density Lipoprotein (DiI-Ac-LDL) Uptake Assay**

After pre-incubation with serum-free medium containing 0.1% BSA for 24 hours, cells were incubated with 10 μg/ml acetylated low density lipoprotein labeled with 1,1'-dioctadecyl-3,3,3',3'-tetramethylindo-carbocyanine perchlorate (Dil-Ac-LDL) (Alfa Aesar) for 5 hours at 37°C and subsequently fixed with 4% PFA/PBS at 4°C for 20 min after washing three times with PBS.

**Tube Formation Assay**

Tube formation assay was performed as described previously.[2] In brief, vascular endothelia derived from iPSCs (4.0 × $10^4$ cells/well) were seeded onto matrigel-coated (Becton Dickinson) 24-well plates. Cells were incubated for 24 h, and digital images of the tubes that formed were captured. Total tube length was calculated using BZ-II analyzer software (KEYENCE) after three randomly selected fields were examined in each well of a 24-well plate. The total tube length was calculated as the average for the three fields.

**Western Blot Analysis**

Immunoblotting was performed for 10 μg of cell lysate extracted with radioimmune

precipitation assay (RIPA) buffer. The membranes were incubated with two primary antibodies, MMP1 (2 μg/ml, MAB901, R&D Systems) and β-Actin (1:1000, A1978, Sigma-Aldrich), followed by incubation with horseradish peroxidase (HRP)-conjugated secondary antibodies (1:5000, NA931, GE Healthcare). The ECL-PLUS Detection System (GE Healthcare) was used as the substrate for chemiluminescent detection. Blots were visualized and scanned using the ImageQuant LAS 4000 Mini (Fujifilm), and the density of bands was measured with Multi Gauge Ver. 3.0 (Fujifilm).

## Additional references

1 Inoue, S. *et al.* Mutation analysis in PKD1 of Japanese autosomal dominant polycystic kidney disease patients. *Human mutation* **19**, 622-628 (2002).

2 Homma, K. *et al.* Sirt1 plays an important role in mediating greater functionality of human ES/iPS-derived vascular endothelial cells. *Atherosclerosis* **212**, 42-47 (2010).

3 Lanner, F., Sohl, M. & Farnebo, F. Functional arterial and venous fate is determined by graded VEGF signaling and notch status during embryonic stem cell differentiation. *Arteriosclerosis, thrombosis, and vascular biology* **27**, 487-493 (2007).

4 Takahashi, K. *et al.* Induction of pluripotent stem cells from adult human fibroblasts by defined factors. *Cell* **131**, 861-872 (2007).

5 McMahon, A. P. & Bradley, A. The Wnt-1 (int-1) proto-oncogene is required for development of a large region of the mouse brain. *Cell* **62**, 1073-1085 (1990).

6 Kruglyak, L., Daly, M. J., Reeve-Daly, M. P. & Lander, E. S. Parametric and nonparametric linkage analysis: a unified multipoint approach. *American journal of human genetics* **58**, 1347-1363 (1996).

7 Rossetti, S. *et al.* Mutation analysis of the entire PKD1 gene: genetic and diagnostic implications. *American journal of human genetics* **68**, 46-63 (2001).

8 Thomas, R. *et al.* Identification of mutations in the repeated part of the autosomal dominant polycystic kidney disease type 1 gene, PKD1, by long-range PCR. *American journal of human genetics* **65**, 39-49 (1999).

9 Watnick, T. *et al.* Mutation detection of PKD1 identifies a novel mutation common to three families with aneurysms and/or very-early-onset disease. *American journal of human genetics* **65**, 1561-1571 (1999).

10 Watnick, T. J. *et al.* An unusual pattern of mutation in the duplicated portion of PKD1 is revealed by use of a novel strategy for mutation detection. *Human molecular genetics* **6**, 1473-1481 (1997).

11 Li, H. L. *et al.* Precise Correction of the Dystrophin Gene in Duchenne Muscular Dystrophy Patient Induced Pluripotent Stem Cells by TALEN and CRISPR-Cas9. *Stem cell reports* **4**, 143-154 (2015).
